# Skeletal muscle delimited myopathy and verapamil toxicity in SUR2 mutant mouse models of AIMS

**DOI:** 10.15252/emmm.202216883

**Published:** 2023-05-08

**Authors:** Conor McClenaghan, Maya A Mukadam, Jacob Roeglin, Robert C Tryon, Manfred Grabner, Anamika Dayal, Gretchen A Meyer, Colin G Nichols

**Affiliations:** ^1^ Center for the Investigation of Membrane Excitability Diseases, and Department of Cell Biology and Physiology Washington University School of Medicine St. Louis MO USA; ^2^ Center for Advanced Biotechnology and Medicine, and Departments of Pharmacology and Medicine, Robert Wood Johnson Medical School Rutgers University Piscataway NJ USA; ^3^ Department of Pharmacology Medical University of Innsbruck Innsbruck Austria; ^4^ Program in Physical Therapy, Departments of Orthopaedic Surgery, Neurology and Biomedical Engineering Washington University School of Medicine St. Louis MO USA

**Keywords:** ABCC9, AIMS, myopathy, SUR2, verapamil, Genetics, Gene Therapy & Genetic Disease, Musculoskeletal System

## Abstract

*ABCC9*‐related intellectual disability and myopathy syndrome (AIMS) arises from loss‐of‐function (LoF) mutations in the *ABCC9* gene, which encodes the SUR2 subunit of ATP‐sensitive potassium (K_ATP_) channels. K_ATP_ channels are found throughout the cardiovascular system and skeletal muscle and couple cellular metabolism to excitability. AIMS individuals show fatigability, muscle spasms, and cardiac dysfunction. We found reduced exercise performance in mouse models of AIMS harboring premature stop codons in *ABCC9*. Given the roles of K_ATP_ channels in all muscles, we sought to determine how myopathy arises using tissue‐selective suppression of K_ATP_ and found that LoF in skeletal muscle, specifically, underlies myopathy. In isolated muscle, SUR2 LoF results in abnormal generation of unstimulated forces, potentially explaining painful spasms in AIMS. We sought to determine whether excessive Ca^2+^ influx through Ca_V_1.1 channels was responsible for myopathology but found that the Ca^2+^ channel blocker verapamil unexpectedly resulted in premature death of AIMS mice and that rendering Ca_V_1.1 channels nonpermeable by mutation failed to reverse pathology; results which caution against the use of calcium channel blockers in AIMS.

## Graphical abstract

Loss‐of‐function (LoF) mutation of ABCC9 (SUR2) results in ABCC9‐related intellectual disability and myopathy syndrome (AIMS), characterized by fatigability, spasticity, and cramping. Genetically modified mouse models were used to study the muscle‐delimited effects of KATP LoF.

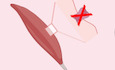

**The paper explained**

**Problem**

Loss‐of‐function (LoF) mutation of *ABCC9*, which encodes the SUR2 subunit of ATP‐sensitive potassium (K_ATP_) channels, causes the rare genetic channelopathy *ABCC9*‐related Intellectual Disability and Myopathy Syndrome (AIMS). SUR2‐containing K_ATP_ channels are expressed throughout the cardiovascular system and in skeletal muscle, and AIMS is characterized by fatigability and muscle dysfunction. How pathology arises in AIMS is incompletely understood, and there is currently no targeted therapy.

**Results**

We show that exercise intolerance and histopathology observed in global SUR2 loss‐of‐function mutant mice is recapitulated in mice expressing K_ATP_ channel dominant‐negative subunits selectively in skeletal muscle—pointing to a skeletal muscle delimited pathological mechanism. Next, we show that loss of SUR2 results in the abnormal development of unstimulated force in isolated muscle subjected to fatiguing stimuli. Previous work has implicated excessive cytosolic calcium in muscle dysfunction resulting from K_ATP_ LoF, but we show that rendering skeletal muscle calcium channels nonpermeable by mutation has no effect on SUR2‐LoF‐induced myopathy. Further, the calcium channel blocker verapamil has unexpected toxic effects in SUR2 LoF mice.

**Impact**

These results, firstly, confirm that skeletal muscle is a key target tissue for future AIMS therapies. Additionally, they indicate that calcium channel blockade, in general, is unlikely to be effective for reversing pathology in the clinic, and that verapamil, specifically, may be harmful.

## Introduction

ATP‐sensitive potassium (K_ATP_) channels, widely expressed in various tissues, couple the cellular metabolic state, and multiple signaling pathways, to the membrane potential and cellular excitability (Nichols, 2006). K_ATP_ channels assemble as octameric complexes of four pore‐forming Kir6 subunits and four regulatory SUR (sulfonylurea receptor) subunits (Shyng & Nichols, 1997; Martin *et al*, 2017; Sung *et al*, 2021), encoded by two pairs of paralogous genes: *ABCC8* (SUR1) and *KCNJ11* (Kir6.2) on human chromosome 11 and *ABCC9* (SUR2) and *KCNJ8* (Kir6.1) on chromosome 12. *ABCC9* transcripts are subject to alternative splicing, with two major splice variants exhibiting distinct tissue‐specific expression: The SUR2A variant is the predominant isoform expressed in striated muscle (where it co‐assembles with Kir6.2) and the SUR2B variant is the predominant isoform in smooth muscle (in co‐assembly with Kir6.1; Inagaki *et al*, 1996; Isomoto *et al*, 1996). Mutations in *ABCC8* and *KCNJ11* result in insulin secretion disorders, while mutations in *ABCC9* or *KCNJ8* cause complex, multiorgan channelopathies with myopathic and cardiac abnormalities (Huang *et al*, 2019). Gain‐of‐function mutations in *KCNJ8* and *ABCC9* cause the rare heritable disorder Cantu Syndrome, while loss‐of‐function (LoF) mutation of *ABCC9* was recently identified as the cause of a novel syndrome, *ABCC9*‐related intellectual disability and myopathy syndrome (AIMS; Harakalova *et al*, 2012; Brownstein *et al*, 2013; Smeland *et al*, 2019). Notably, there is no targeted therapy for AIMS and a better understanding of the pathophysiological mechanisms arising from SUR2 LoF is required to identify potential therapeutic approaches.

AIMS was first identified in two Norwegian families, both of which exhibited conserved facial dysmorphology, mild intellectual disability, impairment in strength and balance, and fatigability with painful muscle stiffness after exercise (Smeland *et al*, 2019). Cardiac systolic dysfunction was observed in older adult subjects. Affected subjects in both families were found to be homozygous for a splice‐site mutation which results in the in‐frame exclusion of exon 8 from mRNA transcripts, leading to the deletion of 52 amino acids (p.Ala389_Gln440del) within the transmembrane domain 1 (TMD1) of the SUR2 protein. Exon 8 deletion in recombinant K_ATP_ channels resulted in complete loss‐of‐function (Smeland *et al*, 2019). Since *ABCC9*‐encoded SUR2 is required for K_ATP_ channel activity in cardiac, smooth, and skeletal muscle (Chutkow *et al*, 2001, 2002), AIMS subjects are expected to lack functional K_ATP_ channels in all muscles. Notably, there is no targeted therapy for AIMS and a better understanding of the pathophysiological mechanisms arising from SUR2 LoF is required to identify potential therapeutic approaches.

Previously, exercise intolerance, skeletal muscle damage, cardiac dysfunction, and vascular abnormalities have all been reported in global SUR2 deficient mice (Chutkow *et al*, 2002; Stoller *et al*, 2009; Smeland *et al*, 2019). As K_ATP_ channels in cardiomyocytes and smooth muscle are required for normal cardiovascular function, it has been suggested that K_ATP_ LoF in these tissues might underlie exercise intolerance and fatigability (Tong *et al*, 2006; Stoller *et al*, 2009). In cardiac muscle, K_ATP_ activation is associated with ischemic preconditioning and protection in conditions of sympathomimetic stress and increased workload (Zingman *et al*, 2002; Gumina *et al*, 2003; Kane *et al*, 2006). K_ATP_ activation in vascular smooth muscle has been implicated in exercise hyperemia and in the regulation of skeletal muscle O_2_ delivery (Holdsworth *et al*, 2015; Colburn *et al*, 2020). Therefore, it is conceivable that the clinically observed fatigability reported in AIMS is of cardiovascular origin. However, global knockout of Kir6.2 (the major pore‐forming subunit of striated muscle K_ATP_ channels) results in contractile dysfunction in isolated skeletal muscle (Gong *et al*, 2003; Cifelli *et al*, 2007, 2008), and thus, pathology in AIMS might alternatively arise from skeletal muscle intrinsic mechanisms.

Interestingly, skeletal muscle from Kir6.2‐null mice exhibits abnormal elevations in cytosolic calcium (Ca^2+^) when subjected to fatiguing stimuli and the phenylalkylamine Ca^2+^ channel blocker, verapamil, has been reported to reverse contractile dysfunction arising from K_ATP_ channel LoF in isolated skeletal muscle (Cifelli *et al*, 2008; Selvin & Renaud, 2015). This may point toward repurposing Ca^2+^ channel blockers for AIMS. We thus sought to determine the cellular origin of the SUR2 loss‐of‐function‐induced myopathy and to investigate the effect of verapamil for reversing pathology. We find that loss of K_ATP_ function in skeletal muscle reconstitutes the major myopathic features observed in global SUR2 LoF mice. Surprisingly, systemic verapamil administration results in sudden death in these mice. Using ncDHPR mice, in which skeletal muscle (Ca_V_1.1) Ca^2+^ channels are rendered nonpermeable to Ca^2+^ by point mutation (Dayal *et al*, 2017, 2021), we show that excessive Ca^2+^ influx through Ca_V_1.1 is not responsible for myopathy arising from K_ATP_ LoF which argues against the use of Ca^2+^ channel blockers (CCBs) for treatment of AIMS symptoms.

## Results

**Figure 1:**
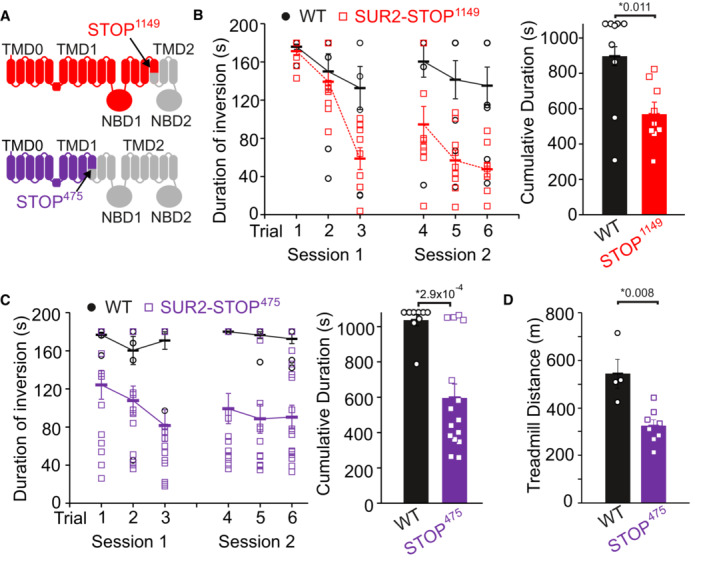
Exercise intolerance in SUR2‐STOP mice ATwo SUR2‐STOP lines were generated with CRISPR‐Cas9‐mediated indel mutations in *ABCC9*. SUR2‐STOP^1149^ mice (as previously described (Smeland *et al*, 2019)) and SUR2‐STOP^475^ in which a 59‐base deletion in exon 9 causes a frameshift and premature stop codon at amino acid position 475 (see Materials and Methods).B, CPerformance in the multitrail inverted screen test is impaired in (B) SUR2‐STOP^1149^ (WT *n* = 9 [3 male; 6 female]; STOP *n* = 9 [3 male; 6 female]; *P* = 0.011 according to Mann–Whitney *U* test) and (C) SUR2‐STOP^475^ mice (WT *n* = 9 [4 male; 5 female]; STOP *n* = 15 [5 male; 10 female]; *P* = 2.9 × 10^−4^ according to Mann–Whitney *U* test).DTreadmill exercise tolerance is also reduced in SUR2‐STOP^475^ mice (WT *n* = 4 [2 male; 2 female]; STOP *n* = 8 [4 male; 4 female]; *P* = 0.008 according to Mann–Whitney *U* test). Treadmill workload tolerated (see Materials and Methods) was also reduced (WT 18.1 ± 2.2 vs. STOP^475^ 9.2 ± 0.9 J; *P* = 0.008 from Man Whitney *U* test), mean mouse body weight was WT 22.1 ± 0.9 vs. STOP^475^ 21.9 ± 0.6 g. Data information: Bars show mean (± SEM) with individual biological replicates shown as circles/squares. * denotes *P*‐value < α = 0.05 for all panels. Source data are available online for this figure.

We first confirmed our previous findings that loss of SUR2 function results in reduced muscle performance of SUR2‐STOP mice (in which a CRISPR/Cas9‐introduced indel mutation resulted in a premature stop codon at position 1149, SUR2‐STOP^1149^; Smeland *et al*, 2019), using a multitrial inverted screen test (Fig 1A and B). We now further confirm this phenotype in a second CRISPR/Cas9 mutant mouse line in which a different indel mutation results in an alternative premature stop codon (SUR2‐STOP^475^; Fig 1A and C). These mice also exhibited reduced performance in treadmill exercise tolerance tests (Fig 1D). Thus, two distinct genetic mouse models of global SUR2 loss‐of‐function recapitulate the impaired exercise tolerance that is observed in human AIMS subjects.

**Figure 2:**
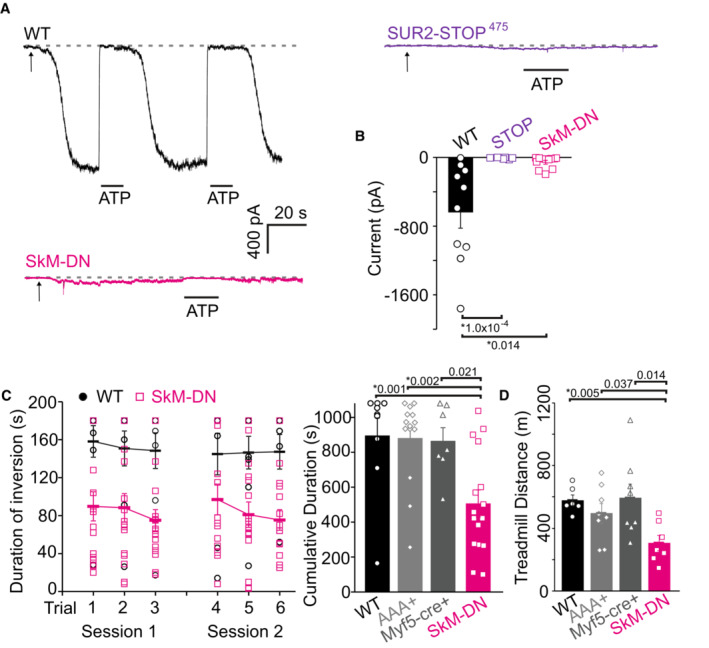
Effect of K_ATP_ LoF in skeletal muscle AExample traces of inside‐out patch clamp recordings of acutely isolated FDB myofibers of WT (*black*, *top left*), SUR2‐STOP^475^ (*purple*, *top right*), and skeletal muscle dominant‐negative (SkM‐DN; *magenta*, *bottom left*) mice. Patches were voltage clamped at −50 mV and excised at time points indicated by arrows. ATP (5 mM) was administered to the cytoplasmic face of membrane patches as indicated by horizontal bars. Dashed lines denote the zero K_ATP_ current level.BMaximal ATP‐sensitive current levels from excised patches. Bars show mean (± SEM) with individual recordings from each myofiber shown as dots/squares (cells from ≥ 3 mice; WT *n* = 10 recordings; STOP *n* = 8; SkM‐DN *n* = 11; Dunn's test following Kruskal–Wallis omnibus test *P* = 1.0 × 10^−4^ for WT vs. STOP and 0.014 for WT vs. SkM‐DN; * denotes *P*‐value < adjusted α = 0.0167).C
*Left*—Performance of WT and SkM‐DN mice in the multi‐trial inverted screen test. *Right—*Cumulative performance across six trials for WT (black circles), single‐transgenic mice carrying either the Kir6.1‐AAA transgene alone (AAA^+^; light gray diamonds) or the Myf5‐Cre transgene alone (Myf5‐cre; dark gray triangles), and double‐transgenic SkM‐DN mice (magenta squares) (WT *n* = 9 [6 male; 3 female]; AAA+ *n* = 14 [10 male; 4 female]; Myf5‐cre *n* = 7 [5 male; 2 female]; SkM‐DN *n* = 16 [12 male; 4 female]; Dunn's test *P* = 0.001 for WT vs. SkM‐DN, 0.002 for AAA+ vs. SkM‐DN, and 0.021 for Myf5‐cre vs. SkM‐DN following Kruskal–Wallis omnibus test; * denotes *P*‐value < adjusted α = 0.0083). Bars show mean (± SEM) with individual mouse performance shown as dots/diamonds/triangles/squares.DDistance traveled in treadmill exercise tolerance tests (all male; WT *n* = 6; AAA+ *n* = 8; Myf5‐cre *n* = 8; SkM‐DN *n* = 7; Dunn's test following Kruskal–Wallis omnibus test *P* = 0.005 for WT vs. SkM‐DN, 0.037 for AAA+ vs. SkM‐DN, and 0.014 for Myf5‐cre vs. SkM‐DN; * denotes *P*‐value < adjusted α = 0.0083). Treadmill workload tolerated was also reduced (WT 23.6 ± 2.3 vs. SkM‐DN 10.1 ± 1.8 J; *P* = 0.0017 from Dunn's test), mean mouse body weight was WT 28.9 ± 1.6, AAA+ 26.6 ± 0.9, Myf5‐cre 25.3 ± 0.8, SkM‐DN 24.2 ± 0.8 g; no significant differences in body weights were observed, * denotes *P* from Dunn's test following Kruskal–Wallis omnibus test < adjusted α = 0.0083 for all comparisons. Bars show mean (± SEM) with individual mouse performance shown as dots/diamonds/triangles/squares. Source data are available online for this figure.

To determine the direct consequences of SUR2 LoF on native skeletal muscle K_ATP_ channels, we recorded excised inside‐out patch‐clamped currents from acutely isolated flexor digitorum brevis (FDB) myofibers. ATP‐sensitive currents were prominent in patches from WT myofibers but completely absent in patches from SUR2‐STOP^475^ myofibers, confirming that functional expression of K_ATP_ in skeletal muscle is dependent on SUR2 (Fig 2A and B).

K_ATP_ function in either or both skeletal muscle and the cardiovascular system could potentially underlie impaired exercise tolerance of global SUR2‐STOP mice. To distinguish between these possibilities, we used a targeted genetic approach to knock down K_ATP_ channel activity specifically in skeletal muscle. Mice expressing Cre‐recombinase under control of the skeletal muscle Myf5 promoter (Tallquist *et al*, 2000) were crossed with mice carrying inducible dominant‐negative mutant *KCNJ8* (Kir6.1) transgenes. Cre‐mediated recombination then results in overexpression of dominant‐negative mutant Kir6.1 subunits that contain a triple‐alanine substitution in the selectivity filter (Kir6.1‐AAA), which hetero‐multimerize with native Kir6 subunits to generate nonfunctional channels (Malester *et al*, 2007; Li *et al*, 2013; McClenaghan *et al*, 2019). In FDB myofibers of resultant double‐transgenic skeletal muscle dominant‐negative (SkM‐DN) mice, functional K_ATP_ channel activity was markedly reduced, as shown in Fig 2A and B. Strikingly, this selective knockdown of K_ATP_ in skeletal muscle resulted in the same exercise intolerance as that observed in the global SUR2‐STOP mice in both the inverted screen and treadmill tests (Fig 2C and D).

In combination, therefore, these results show that SUR2 function is necessary for maintaining physical performance and that loss‐of‐function of K_ATP_ in skeletal muscle is the likely cause of the exercise intolerance reported in AIMS subjects.

**Figure 3:**
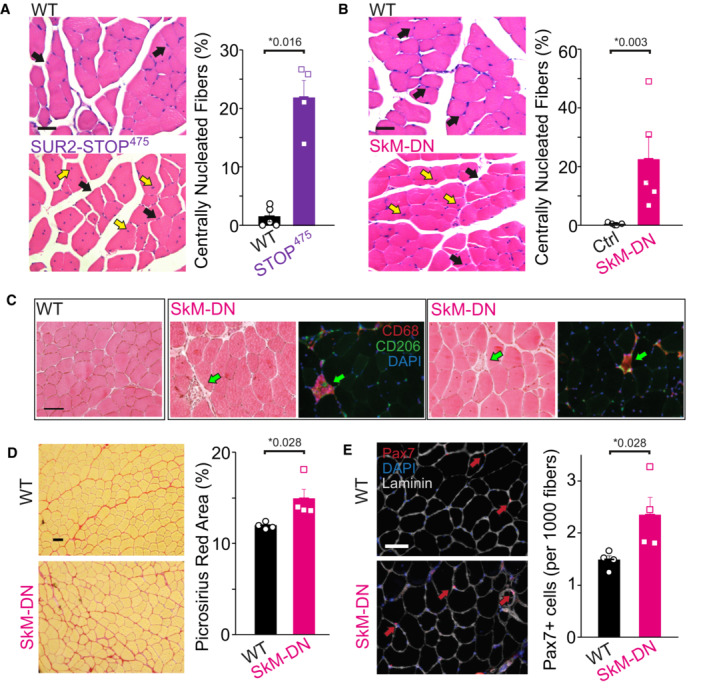
Histopathology due to skeletal muscle K_ATP_ LoF A
*Left—*Example of HE‐stained tibialis anterior (TA) muscles from WT and SUR2‐STOP^475^ mice. Black arrows identify peripheral nuclei and yellow arrows indicate centrally nucleated fibers. *Right—*Quantification of centrally nucleated fibers. Bars show mean (± SEM) with individual biological replicates shown as dots/squares (WT *n* = 5; STOP *n* = 4; *P* = 0.016 according to Mann–Whitney *U* test).B
*Left—*Example of HE‐stained TA muscles from control and SkM‐DN mice. Black arrows identify peripheral nuclei and yellow arrows indicate centrally nucleated fibers. *Right—*Quantification of centrally nucleated fibers. Bars show mean (± SEM) with individual biological replicates shown as dots/squares (Control *n* = 7 [comprising 2 × WT, 3 × Myf5+‐cre, and 2 × AAA+ littermate controls]; SkM‐DN *n* = 5; *P* = 0.002 according to Mann–Whitney *U* test).CExample of HE‐stained WT TA muscle (*left*) and two examples of HE‐stained SkM‐DN TA muscle alongside immunolabeling showing the presence of CD68^+^/CD206^+^ (macrophage marker) cells indicated by green arrows.D
*Left—*Picrosirius red stained TA muscle from WT and SkM‐DN mice. *Right—*Quantification of red stained area (WT *n* = 4; SkM‐DN *n* = 4; *P* = 0.028 according to Mann–Whitney *U* test). Bars show mean (± SEM) with individual measurements shown as dots/squares.E
*Left—*TA muscle for WT and SkM‐DN mice showing Pax7 positive immunolabeled satellite cells (red arrows). *Right—*Quantification of Pax7^+^ cells (WT *n* = 4; SkM‐DN *n* = 4; *P* = 0.028 according to Mann–Whitney *U* test). Bars show mean (± SEM) with individual measurements shown as dots/squares. Data information: * denotes *P*‐value < α = 0.05 for all panels. Scale bars show 50 μm in each panel. Source data are available online for this figure.

In agreement with previous studies of SUR2‐null mice (Stoller *et al*, 2009), we observed a significant increase in centrally nucleated skeletal muscle myofibers in sedentary SUR2‐STOP mice, reflective of increased myofiber damage and regeneration (Fig 3A). In these global knockout mice, this myofiber histopathology could again conceivably arise from intrinsic skeletal muscle dysfunction or from impaired cardiovascular function and blood supply. However, we also found a marked increase in centrally nucleated myofibers in SkM‐DN mice, again suggesting that the myopathy arises due to intrinsic loss of K_ATP_ in skeletal muscle (Fig 3B). Further histological analysis revealed fibrosis in SkM‐DN muscle and the presence of necrotic fibers, marked by macrophage infiltration, which was absent in WT muscle (Fig 3C and D). In addition, increased Pax7 positive satellite cell numbers were observed in SkM‐DN muscle, consistent with injury response (Hardy *et al*, 2016; Fig 3E). Taken together, these data demonstrate that loss of functional skeletal muscle K_ATP_ channels results in impaired exercise tolerance and histopathological markers of myopathy.

**Figure 4:**
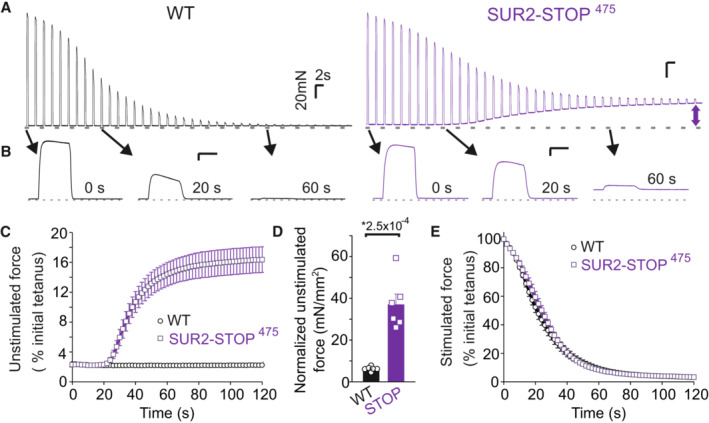
Effect of SUR2 LoF on isolated skeletal muscle contractility AExample traces showing force generation in isometric contractility studies of isolated EDL muscle from WT (left, black) and SUR2‐STOP^475^ (right, purple) mice. Muscle was stimulated every 2 s. Scale bars show 20 mN (*y*‐axis) and 2 s (*x*‐axis).BEnlarged representations of the 1^st^ (at 0 s), 10^th^ (20 s) and 30^th^ (60 s) contractions, identified in concatenated traces in (A) by arrows. Scale bars show 20 mN (*y*‐axis) and 0.4 s (*x*‐axis).CAverage unstimulated force (taken prior to each stimulation) for WT and SUR2‐STOP^475^ myofibers across 60 stimulations. Unstimulated force was normalized to the peak tetanic force generated by the first stimulation. Error bars show SEM (*n* = 6 for both groups).DSummary of unstimulated force (normalized by physiological cross‐sectional area) at 60 s for each experiment. Bars show mean ± SEM. (WT *n* = 6 [3 male; 3 female]; STOP *n* = 6 [3 male; 3 female]; *P* = 2.5 × 10^−4^ according to Mann–Whitney *U* test). * denotes *P*‐value < α = 0.05.EAverage peak stimulated tetanic force for WT and SUR2‐STOP^475^ myofibers (*n* = 6 for both groups). Data shown as mean (± SEM). Source data are available online for this figure.

K_ATP_ channels likely contribute little to the determination of the resting membrane potential in resting muscle, but in fatiguing stimuli the activation of functional K_ATP_ channels protects isolated myofibers from excessive membrane depolarization, cytosolic Ca^2+^ overload, and abnormal development of unstimulated tension (Cifelli *et al*, 2007, 2008; Zhu *et al*, 2014; Selvin & Renaud, 2015). Loss of these myoprotective effects might thus underlie the painful cramping and fatigability reported in AIMS patients. To further determine the effects of loss of SUR2 on muscle performance, we performed contractility measurements in isolated extensor digitorum longus (EDL) muscles from SUR2‐STOP^475^ mice and control littermates. Consistent with previous findings from Kir6.2‐null mice, which also lack K_ATP_ channels in skeletal muscle (Gong *et al*, 2003; Cifelli *et al*, 2007), extensor digitorum longus (EDL) muscles from SUR2‐STOP^475^ mice generate marked unstimulated force in isometric contractility studies when subjected to a repeated tetanic stimulation protocol that results in gradual development of fatigue (Fig 4A–D). In contrast, no major effects on the rate of fatigue were observed between WT and SUR2‐STOP EDL fibers (Fig 4E).

**Figure 5:**
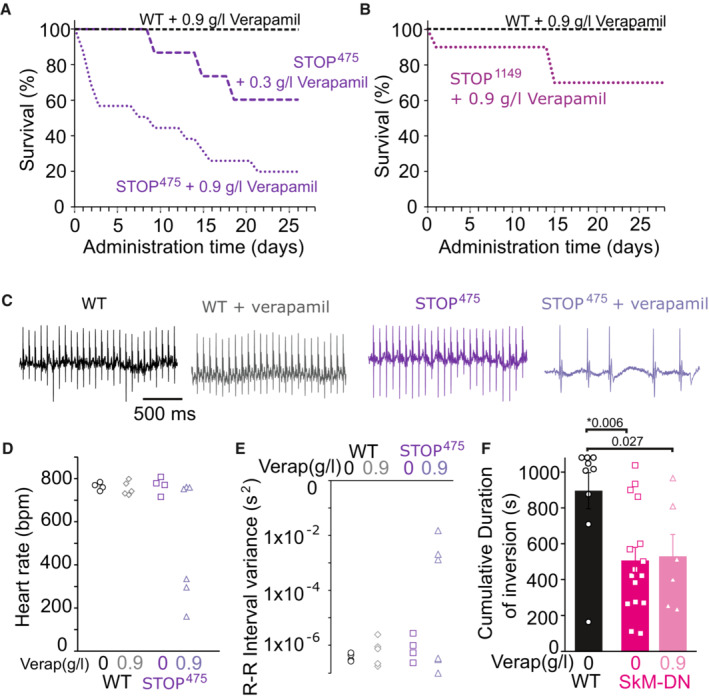
Effects of verapamil administration in K_ATP_‐deficient mice ASurvival of WT and SUR2‐STOP^475^ mice administered with verapamil in drinking water (*n* = 7 [4 male, 3 female] for WT administered 0.9 g/l verapamil; *n* = 14 [8 male, 6 female] for SUR2‐STOP^475^ administered 0.9 g/l verapamil; *n* = 6 [3 male, 3 female] for SUR2‐STOP^475^ administered 0.3 g/l verapamil). Verapamil‐induced deaths were observed in both male and female mice (7 of 8 male and 4 of 6 female SUR2‐STOP^475^ mice died within 28 days of 0.9 g/l verapamil).BSurvival of WT and SUR2‐STOP^1149^ mice administered with verapamil in drinking water (*n* = 10 [3 male, 7 female] for SUR2‐STOP^1149^ mice administered 0.9 g/l verapamil).CFootpad ECG recordings from WT and SUR2‐STOP^475^ mice either without or with 0.9 g/l verapamil administration.DHeart rates calculated from ECG recordings from WT and SUR2‐STOP mice administered with no verapamil (0 Verap) or 0.9 g/l verapamil in drinking water.EVariance in interval between R spikes in QRS complex from footpad ECG recordings.FCumulative durations of inversion in multitrial inverted screen test for WT mice (black circles) and SkM‐DN mice without (magenta squares) or with 0.9 g/l verapamil (pink triangles) administration. Data from WT and SkM‐DN without verapamil same as shown in Fig 2C. Bars show mean ± SEM (WT 0 verapamil *n* = 9 [6 male; 3 female]; SkM‐DN 0 verapamil *n* = 16 [12 male; 4 female]; SkM‐DN with 0.9 g/l verapamil *n* = 6 [4 male; 2 female]; Dunn's test *P* = 0.006 for WT 0 verapamil vs. SkM‐DN 0 verapamil and 0.027 for WT 0 verapamil vs. SkM‐DN 0.9 g/l verapamil following Kruskal–Wallis omnibus test; * denotes *P*‐value < adjusted α = 0.017). Source data are available online for this figure.

In previous studies of isolated K_ATP_‐deficient FDB myofibers, the phenylalkylamine Ca^2+^ channel blocker (CCB) verapamil was shown to protect against the development of unstimulated tension (Cifelli *et al*, 2008; Selvin & Renaud, 2015). This points to the possibility of repurposing such drugs for AIMS. To determine the effects on K_ATP_ LoF‐induced pathology, we administered verapamil in drinking water (0.3–0.9 g/l for > 3 weeks). Surprisingly, premature death was observed within days of administering verapamil to both SUR2‐STOP^475^ and SUR2‐STOP^1149^ mice, while WT littermates were unaffected (Fig 5A and B). This unexpected result indicates that the global loss of K_ATP_ somehow predisposes to verapamil‐induced toxicity. Prior to death, marked bradycardia and arrhythmia (evidenced by increased variance in the R‐R interval in footpad ECG recordings) were observed (Fig 5C–E), which might point toward cardiotoxicity. Notably, SkM‐DN mice all survived verapamil administration, although the drug was without obvious beneficial effects on performance in the inverted screen test (Fig 5F).

Clearly, increased mortality in verapamil‐administered SUR2‐STOP mice suggests that the drug should be avoided in AIMS individuals. Verapamil‐mediated reversal of abnormal unstimulated force in K_ATP_‐null myofibers has previously been suggested to arise due to block of Ca^2+^ influx through skeletal muscle voltage‐gated Ca^2+^ channels (VGCC; Selvin & Renaud, 2015), and understanding the complex consequences of K_ATP_ LoF is necessary to develop targeted therapies for AIMS. Ca^2+^ influx through the skeletal muscle VGCC, Ca_V_1.1 (also referred to as the dihydropyridine receptor; DHPR), is not required for normal excitation‐contraction coupling in skeletal muscle (Schredelseker *et al*, 2010; Dayal *et al*, 2017) but could be markedly elevated in conditions of sustained membrane potential depolarization when K_ATP_ channels are absent, and thereby trigger intracellular Ca^2+^ elevation to increase unstimulated force generation (Cifelli *et al*, 2008; Flucher & Tuluc, 2017).

**Figure 6:**
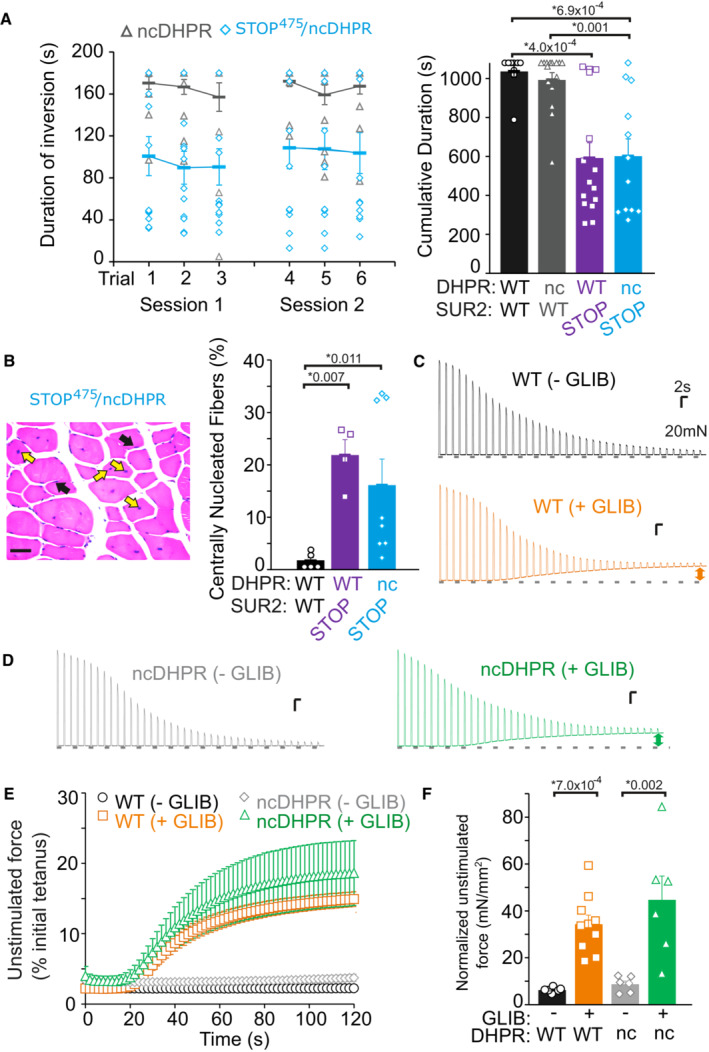
Ca^2+^ influx through Ca_V_1.1 does not affect AIMS‐related pathology A
*Left—*Average performance in the inverted screen test of mice homozygous for the ncDHPR allele alone (ncDHPR) or homozygous for both the ncDHPR and SUR2‐STOP^475^ alleles (STOP^475^/ncDHPR). *Right*—Summary of inverted screen test data. WT and SUR‐STOP^475^ data from experiments shown in Fig 1C. Data shown as mean ± SEM (WT *n* = 9 [4 male; 5 female]; ncDHPR *n* = 15 [male 6; female 9]; STOP^475^ *n* = 15 [5 male; 10 female]; STOP^475^/ncDHPR *n* = 12 [4 male; 12 female]; Dunn's test *P* = 4.0 × 10^−4^ for WT vs. STOP^475^, 6.9 × 10^−4^ for WT vs. STOP^475^/ncDHPR, 0.001 for ncDHPR vs. STOP^475^/ncDHPR verapamil, following Kruskal–Wallis omnibus test). * denotes *P*‐value < adjusted α = 0.0083.B
*Left—*Example of HE‐stained tibialis anterior muscle from STOP^475^/ncDHPR mouse, black arrow shows peripheral nuclei and yellow arrows show central nuclei. *Right—*Quantification of centrally nucleated fibers, data from WT and SUR2‐STOP^475^ mice same as shown in Fig 3A (WT *n* = 5; STOP^475^ *n* = 4; STOP^475^/ncDHPR *n* = 8; Dunn's test *P* = 0.007 for WT vs. STOP^475^ and 0.011 for WT vs. STOP^475^/ncDHPR, following Kruskal–Wallis test, * denotes *P*‐value < adjusted α = 0.067). Scale bar shows 50 μm. Bars show mean (± SEM) with individual measurements shown as dots/squares/triangles.CExample recordings of isolated EDL fibers subjected to fatigue protocol from WT mice in the absence of glibenclamide (top, black) and in the presence of 10 μM glibenclamide (bottom, orange). Scale bars show 20 mN (*y*‐axis) and 2 s (*x*‐axis).DExample recordings from ncDHPR mice in the absence of glibenclamide (left, gray) and in the presence of 10 μM glibenclamide (right, green).EAverage unstimulated force (taken prior to each stimulation) for WT and ncDHPR myofibers in the absence and presence of 10 μM glibenclamide across 60 stimulations. Unstimulated force was normalized to the peak tetanic force generated by the first stimulation. Error bars show SEM with individual biological replicates shown as dots/diamonds/squares/triangles (WT without glibenclamide *n* = 6 [3 male; 3 female]; WT with glibenclamide *n* = 10 [5 male; 5 female]; ncDHPR without glibenclamide *n* = 6 [3 male; 3 female]; ncDHPR with glibenclamide *n* = 6 [3 male; 3 female].FSummary of unstimulated force (normalized by physiological cross‐sectional area) at 60 s for each experiment. Bars show mean ± SEM with individual biological replicates shown as dots/squares/triangles (WT without glibenclamide *n* = 6 [3 male; 3 female]; WT with glibenclamide *n* = 10 [5 male; 5 female]; ncDHPR without glibenclamide *n* = 6 [3 male; 3 female]; ncDHPR with glibenclamide *n* = 6 [3 male; 3 female]; Dunn's test *P* = 7.0 × 10^−4^ for WT without glibenclamide vs. WT with glibenclamide and 0.002 for ncDHPR without glibenclamide vs. ncDHPR with glibenclamide, following Kruskal–Wallis omnibus test; * denotes *P*‐value < adjusted α = 0.0083). Source data are available online for this figure.

To determine whether increased Ca^2+^ influx through Cav1.1 might underlie skeletal muscle pathology in SUR2‐deficient mice, we crossed SUR2‐STOP^475^ mice with nonconductive dihydropyridine receptor (ncDHPR) mice, in which the introduced Ca_V_1.1[N617D] mutation renders the Ca_V_1.1 channel nonpermeable (Dayal *et al*, 2017, 2021). Notably, abolishing Ca_V_1.1 conductances, in mice that were double homozygous for both the SUR2 truncation and the ncDHPR alleles (SUR2‐STOP/ncDHPR mice), had no effect on performance in the inverted screen test: Double homozygous SUR2‐STOP/ncDHPR mice performed as poorly as SUR2‐STOP mice (Fig 6A). These mice also exhibited similarly marked increases in centrally nucleated myofibers (Fig 6B). To examine whether Ca^2+^ influx through Ca_V_1.1 might contribute to the generation of unstimulated force in K_ATP_‐deficient muscle, we also subjected isolated EDL from WT and ncDHPR mice to fatiguing protocols in the absence and presence of the K_ATP_ channel inhibitor glibenclamide. Glibenclamide provoked essentially identical unstimulated force in both WT and ncDHPR muscle (Fig 6C–F). Taken together, these data argue against the possibility that enhanced Ca^2+^ influx through Ca_V_1.1 underlies SUR2‐LoF‐induced myopathy.

## Discussion

ATP‐sensitive potassium channels were first identified in skeletal muscle by Spruce *et al* (1985). Early patch clamp analysis showed that channels were activated by the SUR2‐selective K_ATP_ channel openers cromakalim and pinacidil and inhibited by glibenclamide (Allard & Lazdunski, 1993). This pharmacological profile, along with the observed single channel conductances and later analysis of mRNA transcripts in muscle, suggests a predominant Kir6.2/SUR2 composition, although some fiber‐type specific variations may occur and SUR1 has been proposed to also contribute to K_ATP_ channels in FDB muscle (Tricarico *et al*, 2006). In rats, functional K_ATP_ expression is higher in fast‐twitch (TA < FDB < EDL) than slow‐twitch (soleus) fibers (Tricarico *et al*, 2006). Both channel expression level and subunit composition may vary with development and aging, and expression levels are reduced in hypokalemia (Tricarico & Camerino, 1994; Tricarico *et al*, 1997, 2008). Previous studies of Kir6.2‐KO mice showed essentially complete loss of channel activity, consistent with Kir6.2 acting as the predominant pore‐forming subunit (Miki *et al*, 2002). Here, we show that functional K_ATP_ expression in mouse FDB myofibers is critically dependent on SUR2, further confirming the long‐held postulate that Kir6.2 and SUR2 comprise the predominant skeletal myocyte subunits. If this is also the case in human muscle, AIMS subjects, who are homozygous for SUR2 LoF mutations, would be expected to exhibit complete loss of skeletal myocyte K_ATP_ expression.

K_ATP_ channels in muscle open in metabolically compromising conditions, including in mild exercise (Castle & Haylett, 1987; Light *et al*, 1994; Zhu *et al*, 2014). Pharmacological inhibition of K_ATP_ channels during fatigue results in membrane depolarization, increased ATP depletion, cytosolic Ca^2+^ overload, increased resting tension, and impaired recovery (Gramolini & Renaud, 1997; Matar *et al*, 2000; Gong *et al*, 2003; Cifelli *et al*, 2007, 2008), whereas pharmacological activation enhances the rate of fatigue but preserves ATP and phosphocreatine levels (Matar *et al*, 2000). In this way, K_ATP_ channel activation in muscle serves a myoprotective role, generating an electrical brake that prevents contraction‐dependent energy depletion. Fatiguing stimuli also provoke marked unstimulated force generation in Kir6.2‐null mice (Gong *et al*, 2003; Cifelli *et al*, 2007). Here, we show similar unstimulated contraction in isolated SUR2‐deficient muscle, which may explain the painful spasms experienced by AIMS individuals after exercise—and given the apparently normal fatigue rate in isolated SUR2‐STOP EDL muscle, might also explain the poor inverted screen test and treadmill performance in mice due to increased muscle cramping or impaired relaxation.

Interestingly, exercise intolerance, muscle fatigue, and histopathology are also observed in the rare K_ATP_ channelopathy, Cantú Syndrome, which arises from gain‐of‐function mutations in either *ABCC9* (SUR2) or *KCNJ8* (Kir6.1; Grange *et al*, 2019; Scala *et al*, 2020; Scala *et al*, 2021). Whether this is due to intrinsic effects in skeletal muscle physiology or is due to altered cardiovascular function and/or systemic perfusion remains to be established. Individuals with LoF mutations in *KCNJ11* (Kir6.2) suffer from congenital hyperinsulinism (CHI). Although muscle fatigue is not typically reported, one extended consanguineous family, exhibiting a syndrome of CHI and rhabdomyolysis has been reported in association with the Kir6.2[R34H] mutation (Albaqumi *et al*, 2014). In this case, the CHI was severe, and potentially the loss of Kir6.2‐dependent K_ATP_ was profound: Previous analysis of the R34A mutation renders the channels completely insensitive to the essential activator PIP_2_ (Cukras *et al*, 2002)_._ Why myopathy is not more widely observed in CHI is not clear but could be explained by incomplete loss‐of‐function or possibly some underappreciated contribution of Kir6.1 subunits to skeletal muscle channels.

In demonstrating that the performance defects observed in global SUR2‐STOP mice result from knockdown of skeletal muscle channels specifically, our data point to a fundamental role for skeletal muscle‐delimited K_ATP_ dysfunction in the exercise intolerance observed in AIMS. We thus identify skeletal muscle as a key tissue to target in developing AIMS therapies, even though myocardial and vascular K_ATP_ channels have also been implicated in exercise performance and may also contribute to AIMS fatigability (Tong *et al*, 2006; Holdsworth *et al*, 2015; Colburn *et al*, 2020).

In the case of AIMS, wherein homozygous variants result in major deletions in SUR2 (Smeland *et al*, 2019), attempts at pharmacological recovery of K_ATP_ are likely to be futile, and addressing secondary effects may be necessary for clinical benefit. The phenylalkylamine Ca^2+^ channel blocker (CCB) verapamil has been reported to reverse contractile dysfunction in Kir6.2‐null and glibenclamide‐treated isolated myofibers, both of which exhibit Ca^2+^ overload in fatigue (Cifelli *et al*, 2008; Selvin & Renaud, 2015). We therefore sought to determine whether the drug could reverse AIMS pathology in SUR2‐STOP mice. This is particularly pertinent given that AIMS patients exhibit borderline high blood pressures which might inspire CCB administration (Smeland *et al*, 2019). However, the surprising verapamil‐induced lethality in SUR2‐null mice clearly argues against this. How verapamil results in death in SUR2‐null mice remains to be established, but the bradycardia and arrhythmia that precede death, as well as the survival of SkM‐DN mice, strongly point toward a cardiotoxic effect. Verapamil is known to provoke bradycardia (Wit & Cranefield, 1974), and SUR2 is a component of K_ATP_ channels in both contractile cardiomyocytes and specialized cells of the cardiac conduction system, including nodal cells (Bao *et al*, 2011; Aziz *et al*, 2018a). Since the loss of K_ATP_ in the cardiac conduction system cells prolongs action potential (AP) duration (Aziz *et al*, 2018b), it is tempting to speculate that AP prolongation due to loss of SUR2 function might enhance open‐state block of nodal Ca_V_1.2 channels by the use‐dependent Ca^2+^ channel blocker verapamil, driving bradycardia and arrhythmia. However, other mechanisms, including “off‐target” non‐Ca^2+^ channel blocking effects, could also contribute.

Finally, we sought to determine, via a genetic approach, whether Ca^2+^ influx through the skeletal muscle VGCC, Ca_V_1.1, might be responsible for myopathy in SUR2‐STOP mice. To do so, we used ncDHPR (nonconductive dihydropyridine receptor) mice. These mice harbor the N617D mutation in *CACNA1S*, which renders Ca_V_1.1 channels nonconductive without altering normal excitation‐contraction coupling—which is mediated through the physical linkage of Ca_V_1.1 and sarcoplasmic reticulum ryanodine receptors (Schredelseker *et al*, 2010; Dayal *et al*, 2017, 2021). Exercise intolerance and histopathology were unchanged in SUR2‐STOP mice with ncDHPR mutation, arguing against Ca^2+^ influx through Ca_V_1.1 being responsible for pathology. As further evidence, we also found that unstimulated force generation provoked by glibenclamide was the same in both WT and ncDHPR mice. Previous studies have shown that verapamil can reverse contractile dysfunction in isolated glibenclamide‐treated myofibers (Cifelli *et al*, 2008), but our results suggest this effect must occur independent of Ca_V_1.1 blockade. While our data thus suggest that Ca_V_1.1 blockade is unlikely to be a protective approach in AIMS, it is conceivable that modulation of the voltage‐sensing function of Ca_V_1.1 channels, or modulation elsewhere in the excitation‐contraction coupling cascade, might have clinical benefit.

The present study helps to explain the cellular origin of AIMS‐related pathology: We show that SUR2 is essential for skeletal muscle K_ATP_ function, that the absence of SUR2 results in abnormal unstimulated contraction, and that loss of K_ATP_ specifically in skeletal muscle provokes exercise intolerance. While providing a framework for the future preclinical testing of therapies for AIMS, including *ex vivo* and *in vivo* assays of muscle dysfunction in SUR2‐deficient mice, our results warn against the use of verapamil in AIMS individuals.

## Materials and Methods

### Mouse models and study approval

SUR2‐STOP mice were generated by CRIPSR‐Cas9 genome engineering as previously described and are available for sharing with other investigators (Smeland *et al*, 2019). SUR2‐STOP^1149^ mice carry a premature stop mutation at position 1149 in the transmembrane domain 2 of SUR2, as previously reported (Smeland *et al*, 2019). SUR2‐STOP^475^ mice carry a (NM_021041.2) c.1315_1373del deletion resulting in a major deletion, frameshift, and premature stop codon at position 475 in transmembrane domain 1 of SUR2. This deletion changes the encoded amino acid sequence from 450‐LGSSALVGAAVIVLLAPIQYFIATKL‐475 in the wild‐type sequence to 450‐LHCHEAGGGSEEHSGLFHREVEEDE*‐475 where * indicates a stop codon. Kir6.1‐AAA mice were generated as previously described (Malester *et al*, 2007) and crossed to mice expressing Cre‐recombinase under the Myf5 promoter (Jackson Labs; 007893; Myf5‐Cre or Myf5+; Tallquist *et al*, 2000). ncDHPR mice were generated as previously described (Dayal *et al*, 2017). Mice were genotyped via Transnetyx (Cordova, TN, USA). Male and female adult mice aged 3–5 months were used for all but one experiment and combined for statistical analysis as no significant differences were observed between sexes for any of the phenotypes (all *P*‐values > 0.05 from Mann–Whitney *U* tests for male vs. females of the same genotype). The single exception was for treadmill tests of tissue‐specific dominant‐negative Kir6.1‐AAA expression effects. Here, female Myf5‐Cre mice exhibited worse performance than males (*P* = 0.023 from Mann–Whitney *U* test, male *n* = 8, female *n* = 5), and thus, only male mice were used for all genotypes. Investigators were blinded to genotype at testing.

Studies were performed in compliance with the standards for the care and use of animal subjects defined in the NIH Guide for the Care and Use of Laboratory Animals and were reviewed and approved by the Washington University Institutional Animal Care and Use Committee.

### Exercise tolerance testing in mice

The multitrial inverted screen test was performed as previously reported (Smeland *et al*, 2019) and is designed to test strength and endurance. Briefly, mice were placed on a wire mesh screen (16 squares per 10 cm), which was then inverted leaving mice hanging from the screen. Soft padding was placed underneath mice to protect from harm from falls. The time the mouse remained on the screen over the first 3‐min trial was recorded. The mouse was then subjected to two further trials interspersed by 5‐min rest periods in the home cage. This 3‐trial regime was repeated (for trials 4, 5, and 6) after a 44‐min rest period following trial 3.

Treadmill testing was performed as previously reported (McClenaghan *et al*, 2020). Briefly, after 3 days of habituation to the treadmill apparatus (Columbus Instruments Model Exer3/6 Treadmill) for 10 min per day at 10 m/min (at 10^o^ incline), mice were tested for maximal exercise tolerance on Day 4. Treadmill speed was set to 10 m/min, and speed was increased by 3 m/min every 3 min until exhaustion. Exhaustion was defined as a mouse remaining on the shock grid for five continuous seconds. During habituation and testing, the voltage shock was set at 0.5 mA (2 Hz). The total distance (in meters) covered by each mouse was calculated alongside the total tolerated workload (in Joules) performed. Workload was calculated as the sum of the kinetic (E_K_) and potential (E_P_) energy as previously described (Zingman *et al*, 2002) and according to Work = E_K_ + E_P_; E_K_ = mv^2^/2; and EP = m.g.t.sin(φ), where m is the mouse mass (in kilogram), v is the belt velocity (in meters per second), g is acceleration due to gravity (taken as 9.81 m/s^2^), t is duration of exercise (in seconds), and φ is the angle of incline of the treadmill.

### Electrophysiology

Flexor digitorum brevis (FDB) muscle was dissected from terminally anesthetized mice and digested in 2 mg/ml collagenase type 2 in Dulbecco's modified Eagle's Medium (supplemented with 10% fetal bovine serum and 1% penicillin/streptomycin; DMEM) at 37°C for 1 h in a humidified 95% O_2_/5% CO_2_ incubator. Single myofibers were isolated by gentle trituration of digested tissue through a fire‐polished Pasteur pipette and maintained in DMEM for up to 6 h during recording. Myofibers suspended in DMEM were added to the patch clamp recording chamber and washed with KINT solution containing (in mM): 140 KCl, 10 HEPES, 1 EGTA (pH 7.4 with KOH). Recording electrodes were formed from soda‐lime hematocrit glass (Kimble) with resistances of 1–2 MΩ when filled with KINT solution. Currents were recorded at −50 mV, sampled at 3 kHz, and filtered at 1 kHz using an Axopatch 200B and Digidata 1200 (Molecular Devices). K_ATP_ currents spontaneously developed after patch excision and maximal currents were calculated as the difference between the current in KINT without ATP and KINT containing 5 mM ATP. The data were analyzed using Clampfit (Molecular Devices) and Excel (Microsoft).

### Histology

Tibialis anterior muscle was dissected, fixed in 10% buffered formalin for 24 h, and embedded in paraffin. Sections (8 μm) were cut and stained with hematoxylin and eosin (HE). Stained sections were imaged, and centrally nucleated fibers manually counted. Centrally nucleated fibers and total fiber counts were made from five images from three different sections from each sample. Wild‐type and single‐transgenic mice carrying either the Myf5+‐cre or AAA+ allele alone were combined as littermate controls for SkM‐DN mice in comparisons of centrally nucleated fiber numbers.

Separate tibialis anterior muscles were flash‐frozen in liquid nitrogen‐cooled isopentane and cryosectioned at 10 μm on a cryostat (Leica CM1950). Sections were stained with HE and Picrosirius Red as previously described (Meyer *et al*, 2022). Briefly, sections were fixed in chilled acetone (60 min) followed by Bouin's solution (5 min). Sections were then rinsed under running dH_2_O (10 min) and transferred to freshly made Picrosirius Red solution (1 mg/ml Direct Red 80 in saturated picric acid) for 2 h. Sections were then placed in 0.01 M hydrochloric acid for 5 min followed by dehydration in ethanol, clearance in xylenes and coverslipping. Sirius red area fraction was averaged from two 10× images comprising > 50% of the section area and was quantified by thresholding the red channel with the Huang algorithm in ImageJ (NIH).

Serially cut sections were also fixed in 4% paraformaldehyde for 10 min, permeabilized in 1% Triton‐X for 10 min and immunostained against pan‐macrophage (CD68, Abcam ab955), type 2 macrophage (CD206, Cell Signaling Technologies 24595) and satellite cell (Pax7, Developmental Studies Hybridoma Bank PAX7; counterstained with laminin, Abcam 11575) markers. Pax7 immunostaining required an additional step of antigen retrieval which involved heating slides in Antigen Retrieval Citra (Biogenex; HK086‐5K) for 15 min in a 5 qt steamer (Aurora Housewares). Sections were blocked (1% bovine serum albumin, 5% goat serum, 0.3% Triton‐X) for 1 h and incubated with primary antibodies overnight at 4°C. Appropriate fluorescent secondary antibodies were applied the following day. Pax7‐positive cells were identified by co‐registration of DAPI and Pax7 beneath a laminin‐positive basal lamina and were counted by hand.

### Isolated isometric contractility studies

Extensor digitorum longus (EDL) muscle was dissected from mice under isoflurane anesthesia. Muscle was transferred to the recording chamber (1500A, Aurora Scientific, Aurora, ON, Canada), immersed in Mammalian Ringer's solution which contained (in mM; NaCl 137, KCI 5, CaCl_2_ 2, NaH_2_PO_4_ 1, NaHCO_3_ 24, MgSO_4_ 1, glucose 11, and curare 0.015) maintained at 37°C. The distal tendon was secured at one end to a dual‐mode ergometer (300C‐LR, Aurora Scientific, Aurora, ON, Canada) with the other secured to a stationary pin. Muscles were stimulated with an electrical stimulator (701C, Aurora Scientific, Aurora, ON, Canada) with platinum plate electrodes parallel to the muscle. Peak muscle length was determined by incrementally increasing length and measuring tetanic force generation until it plateaued, and measured as the tendon‐to‐tendon length using an optical reticule mounted in a dissecting microscope. For basal measurements, muscles were incubated in Ringers solution for 10 min between optimal length identification and initiation of the fatigue test. For glibenclamide‐treated muscles, Ringers was exchanged for Ringers supplemented with 10 μM glibenclamide (Millipore‐Sigma) and incubated for 10 min between optimal length identification and initiation of the fatigue test.

The fatigue protocol involved stimulating isometric tetanic contraction by 300 ms trains of 0.3 ms pulses (at 225 Hz) every 2 s. After testing, muscle was cut from sutures and weighed. For conversion to specific forces, measured forces (in mN) were divided by physiological cross‐sectional area (PCSA; calculated as the mass in g × cos(α)/muscle density × fiber length at peak force in mm; where α = angle of pennation, taken as 0.295; and muscle density was taken as 1.06 g/cm^3^). Data were analyzed in MatLab (Mathworks) and Excel (Microsoft). Unstimulated force was measured prior to each stimulation. Stimulated force was calculated as the peak tetanic force minus unstimulated force for each stimulation and was expressed for each stimulation as a percentage of the initial stimulated force.

### Verapamil administration

Mice were administered with verapamil hydrochloride (Cayman Chemicals) mixed in drinking water at either 0.3 or 0.9 g/l, provided *ad libitum*. Dosages were chosen based on previous studies showing blood pressure‐lowering effects, indicating efficacy, and the absence of toxicity in hypertensive rats at 0.9 g/l (Lederballe Pedersen *et al*, 1982). ECG recordings were made from conscious mice placed in a Perspex restraint upon silver footpads connected to a differential amplifier (Model 1700, A‐M Systems) and digitizer (Digidata 1200, Axon Instruments) and recorded on Clampex (Molecular Devices). R‐R intervals were measured manually in Clampfit (Molecular Devices) across 8–10 heartbeats, and variance (the square of R‐R interval standard deviation for heartbeats measured in each mouse) was calculated in Excel (Microsoft).

### Statistics

Statistical analysis was carried out with Microsoft Excel (Real Statistics Resource Pack software, www.real‐statistics.com). Significance values were calculated using either Mann–Whitney *U* tests (when comparing samples from two groups) or Kruskal–Wallis tests followed by pairwise Dunn's tests to account for multiple comparisons (when comparing > 2 groups). Full descriptions of test statistics are including with the source data files.

## Author contributions

**Conor McClenaghan:** Conceptualization; data curation; formal analysis; supervision; funding acquisition; validation; investigation; visualization; writing – original draft; project administration; writing – review and editing. **Maya A Mukadam:** Data curation; formal analysis; investigation; writing – review and editing. **Jacob Roeglin:** Data curation; formal analysis; investigation; writing – review and editing. **Robert C Tryon:** Project administration; writing – review and editing. **Manfred Grabner:** Resources; writing – review and editing. **Anamika Dayal:** Resources; writing – review and editing. **Gretchen A Meyer:** Conceptualization; data curation; formal analysis; investigation; writing – original draft; writing – review and editing. **Colin G Nichols:** Conceptualization; supervision; writing – original draft; writing – review and editing.

In addition to the CRediT author contributions listed above, the contributions in detail are:

CM contributed to conceptualization, data curation, formal analysis, investigation, supervision, and writing; MAM contributed to data curation, formal analysis, and investigation; JR contributed to data curation, formal analysis, and investigation; RCT contributed to project administration; MG and AD contributed to resources and writing; GAM contributed to conceptualization, data curation, formal analysis, investigation, and writing; CGN contributed to conceptualization, supervision, and writing.

## Disclosure and competing interests statement

The authors declare that they have no conflict of interest.

## Supporting information



Source Data for Figure 1

Source Data for Figure 2

Source Data for Figure 3

Source Data for Figure 4

Source Data for Figure 5

Source Data for Figure 6

## Data Availability

No data herein require deposition in a public database.
