# Supplementary figures and images for: Skeletal muscle delimited myopathy and verapamil toxicity in SUR2 mutant mouse models of AIMS
Source: EMBO Mol Med. 2023 May 8;15(6):e16883. doi: 10.15252/emmm.202216883 (PMC10245035; doi:10.15252/emmm.202216883)

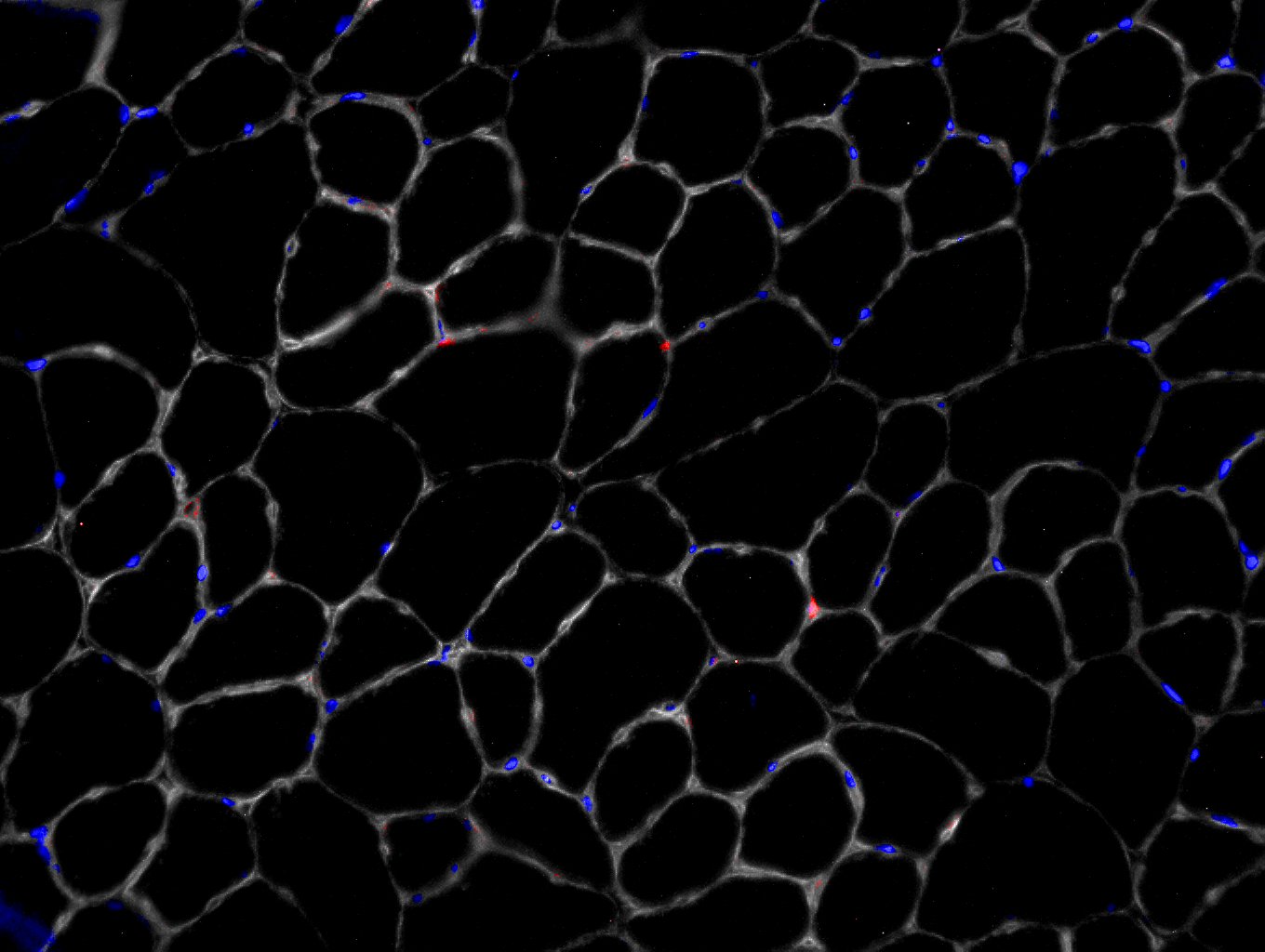

Supplement: Supplementary file 3 — Source Data for Figure 3 [file EMMM-15-e16883-s004.zip › Figure 3/3E/3E WT.png]

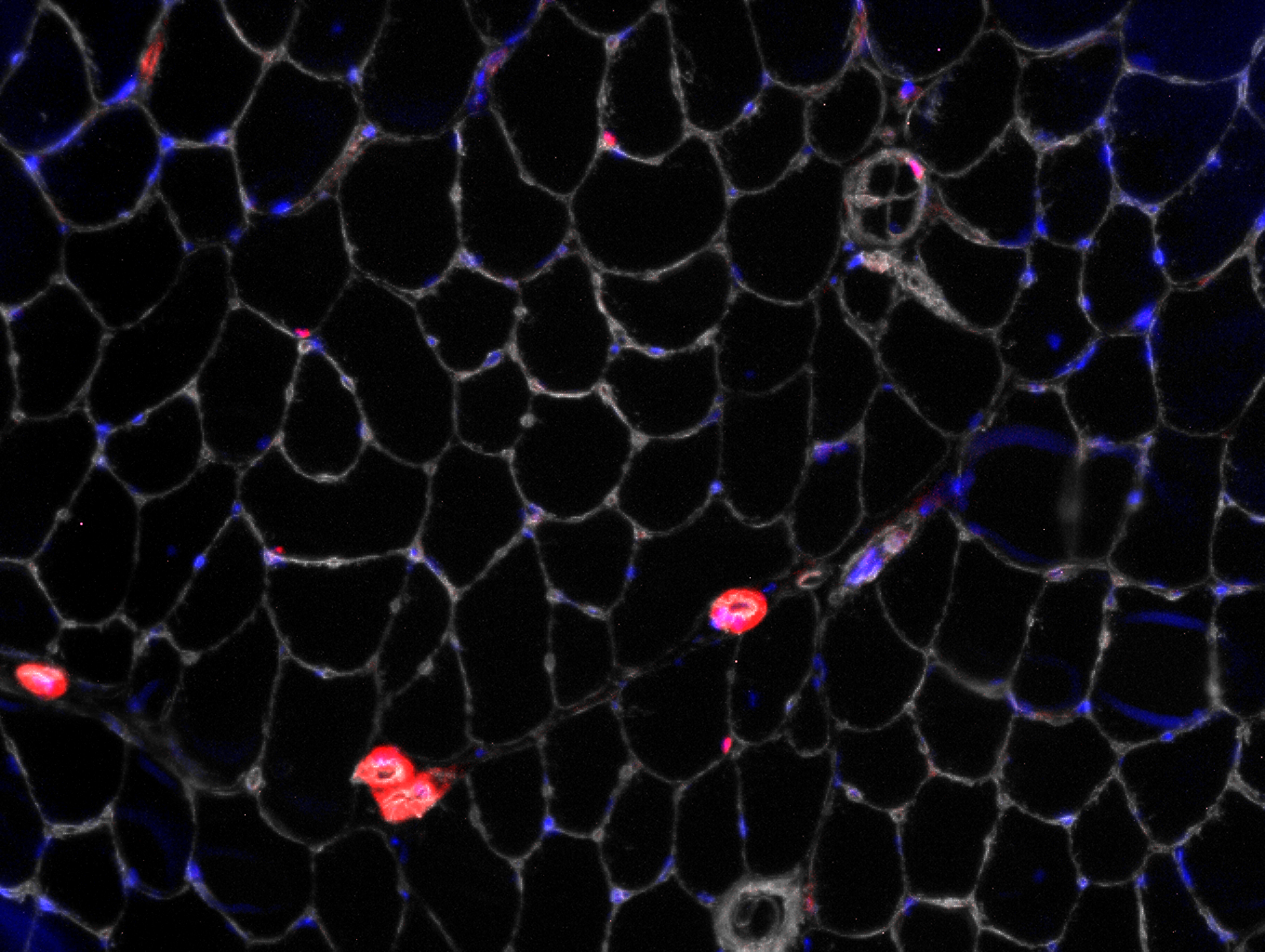

Supplement: Supplementary file 3 — Source Data for Figure 3 [file EMMM-15-e16883-s004.zip › Figure 3/3E/3E SkMDN.png]

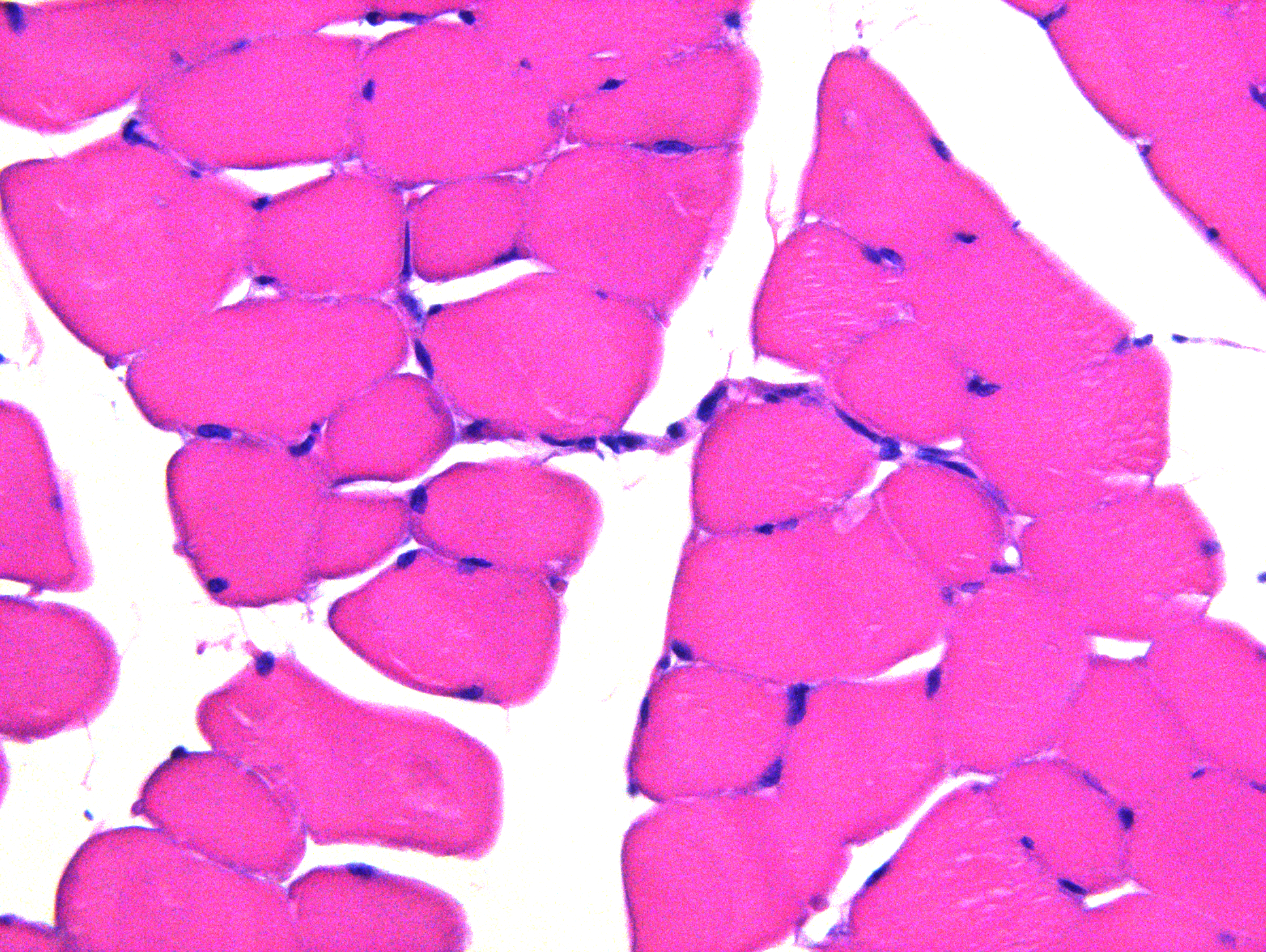

Supplement: Supplementary file 3 — Source Data for Figure 3 [file EMMM-15-e16883-s004.zip › Figure 3/3B/3B WT.tif]

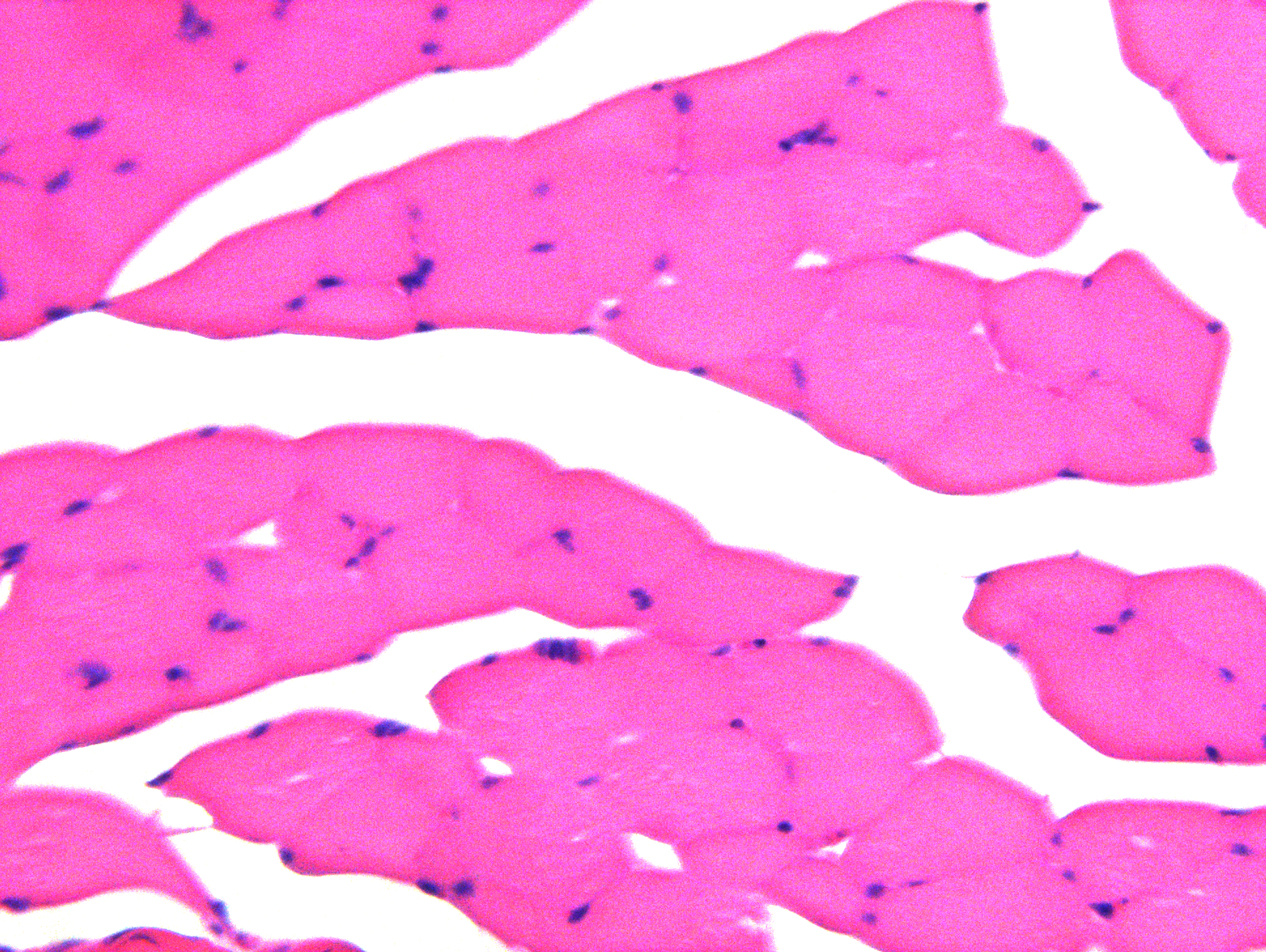

Supplement: Supplementary file 3 — Source Data for Figure 3 [file EMMM-15-e16883-s004.zip › Figure 3/3B/3B SkMDN.tif]

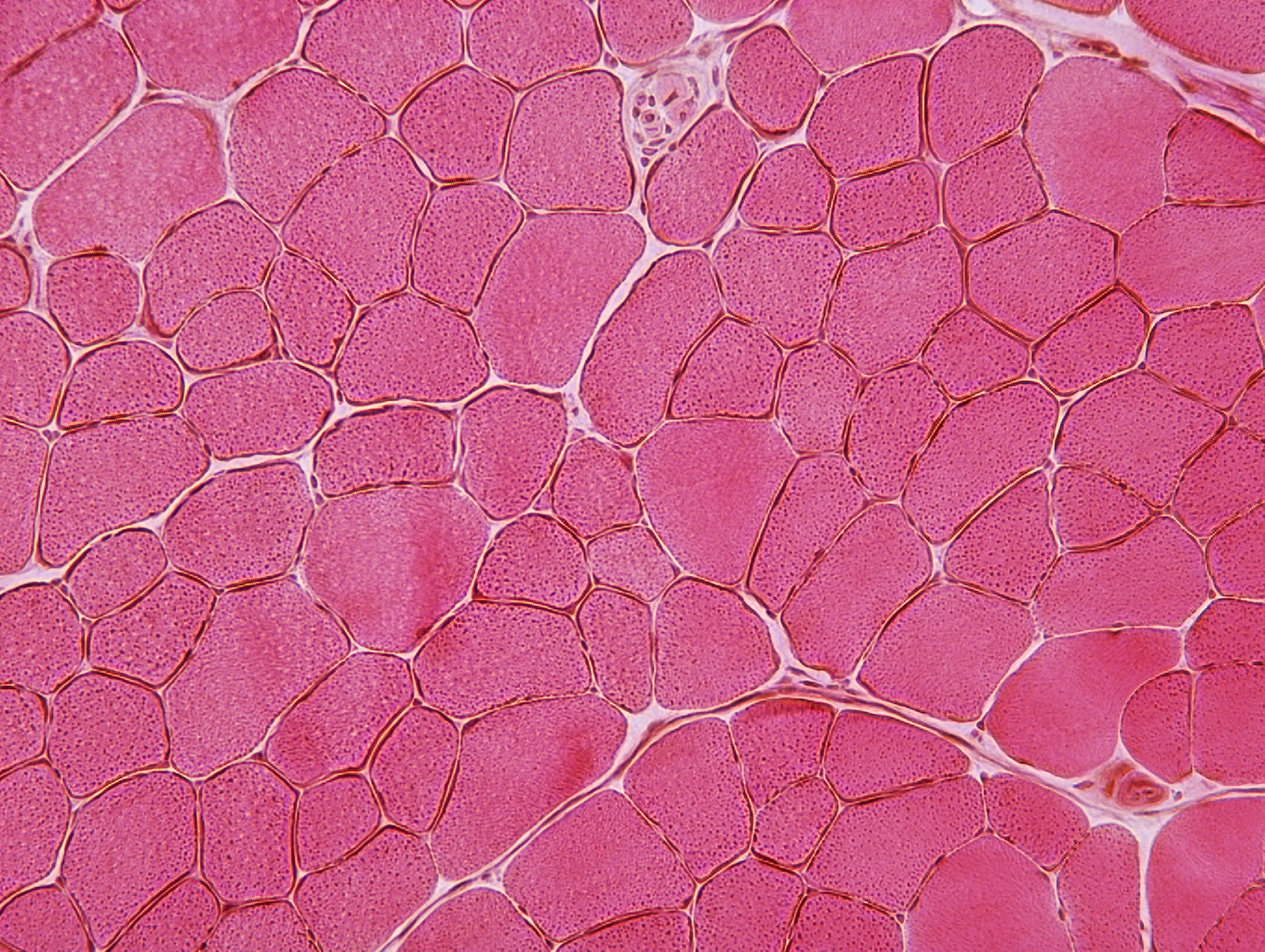

Supplement: Supplementary file 3 — Source Data for Figure 3 [file EMMM-15-e16883-s004.zip › Figure 3/3C/3C WT.tif]

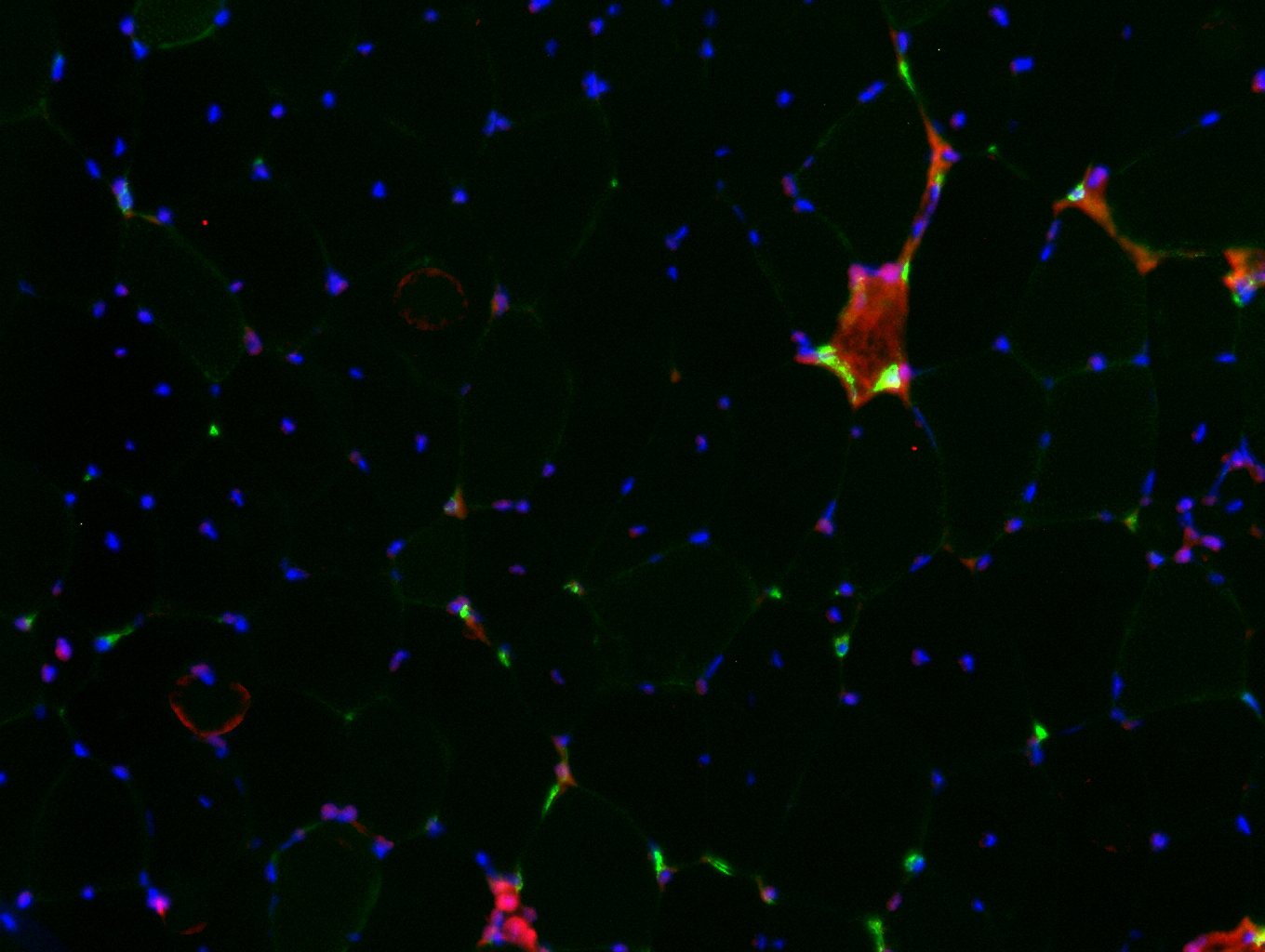

Supplement: Supplementary file 3 — Source Data for Figure 3 [file EMMM-15-e16883-s004.zip › Figure 3/3C/3C SkMDNii.png]

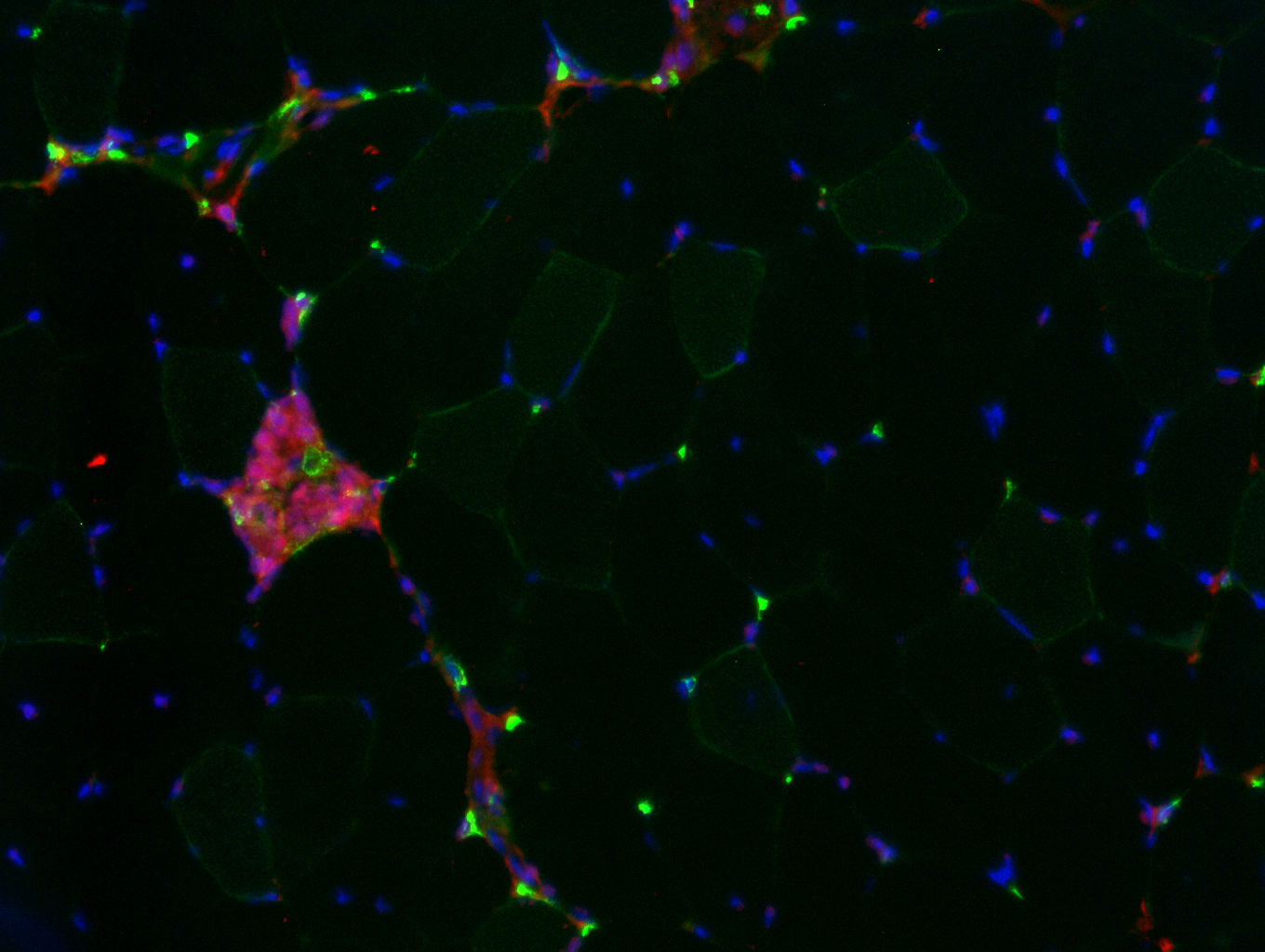

Supplement: Supplementary file 3 — Source Data for Figure 3 [file EMMM-15-e16883-s004.zip › Figure 3/3C/3C SkMDNi.png]

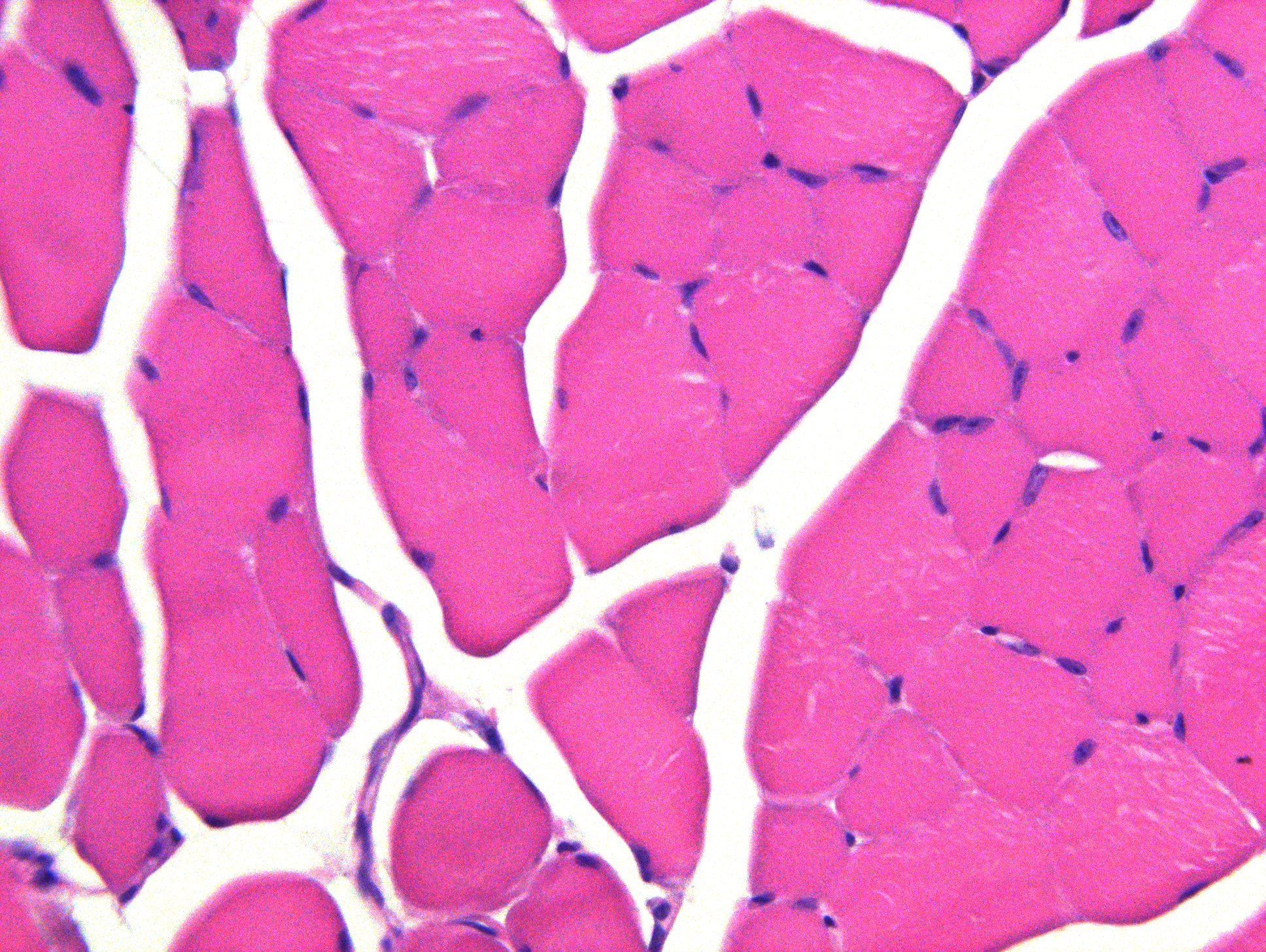

Supplement: Supplementary file 3 — Source Data for Figure 3 [file EMMM-15-e16883-s004.zip › Figure 3/3A/3A WT.jpg]

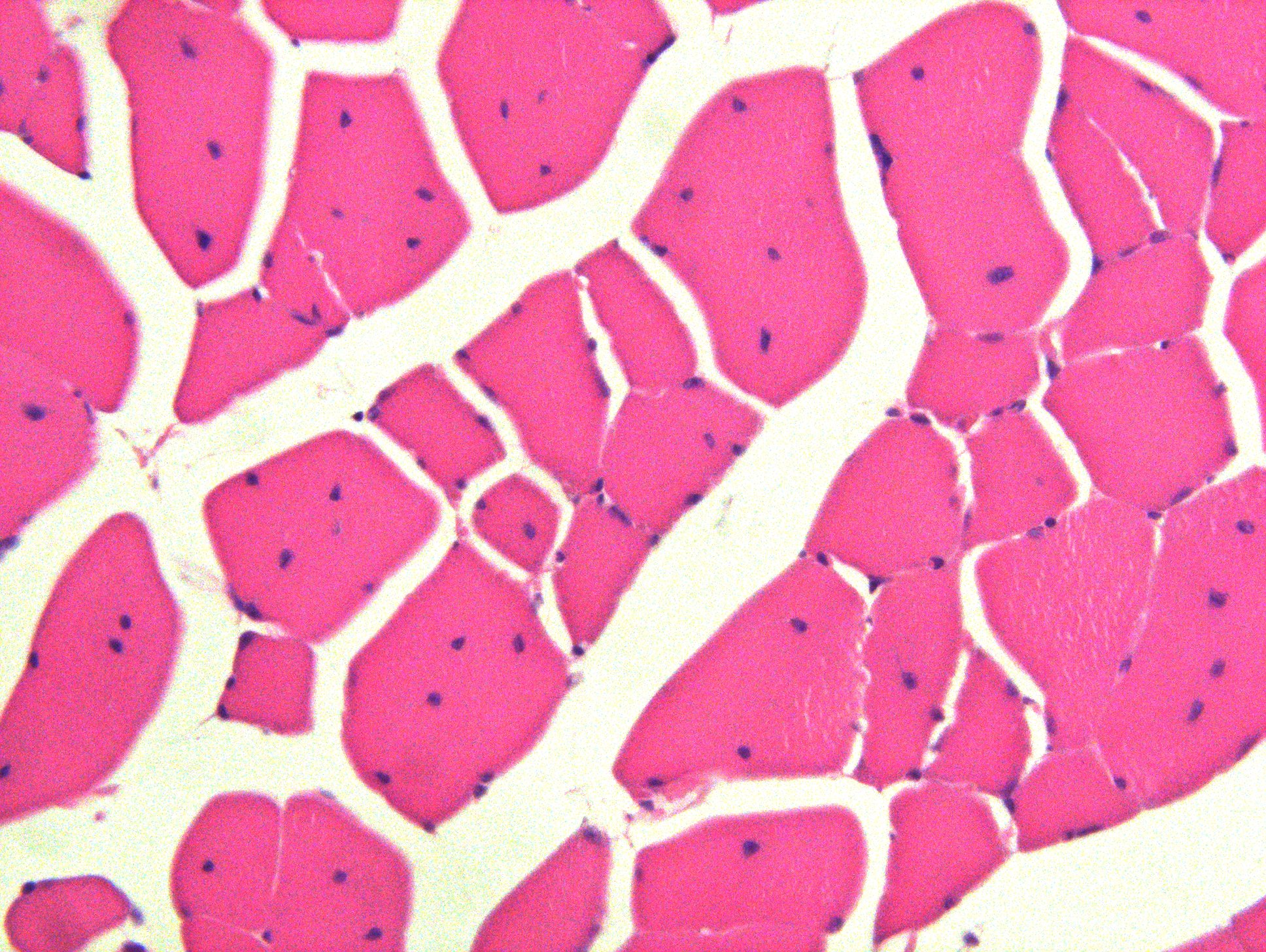

Supplement: Supplementary file 3 — Source Data for Figure 3 [file EMMM-15-e16883-s004.zip › Figure 3/3A/3A STOP475.jpg]

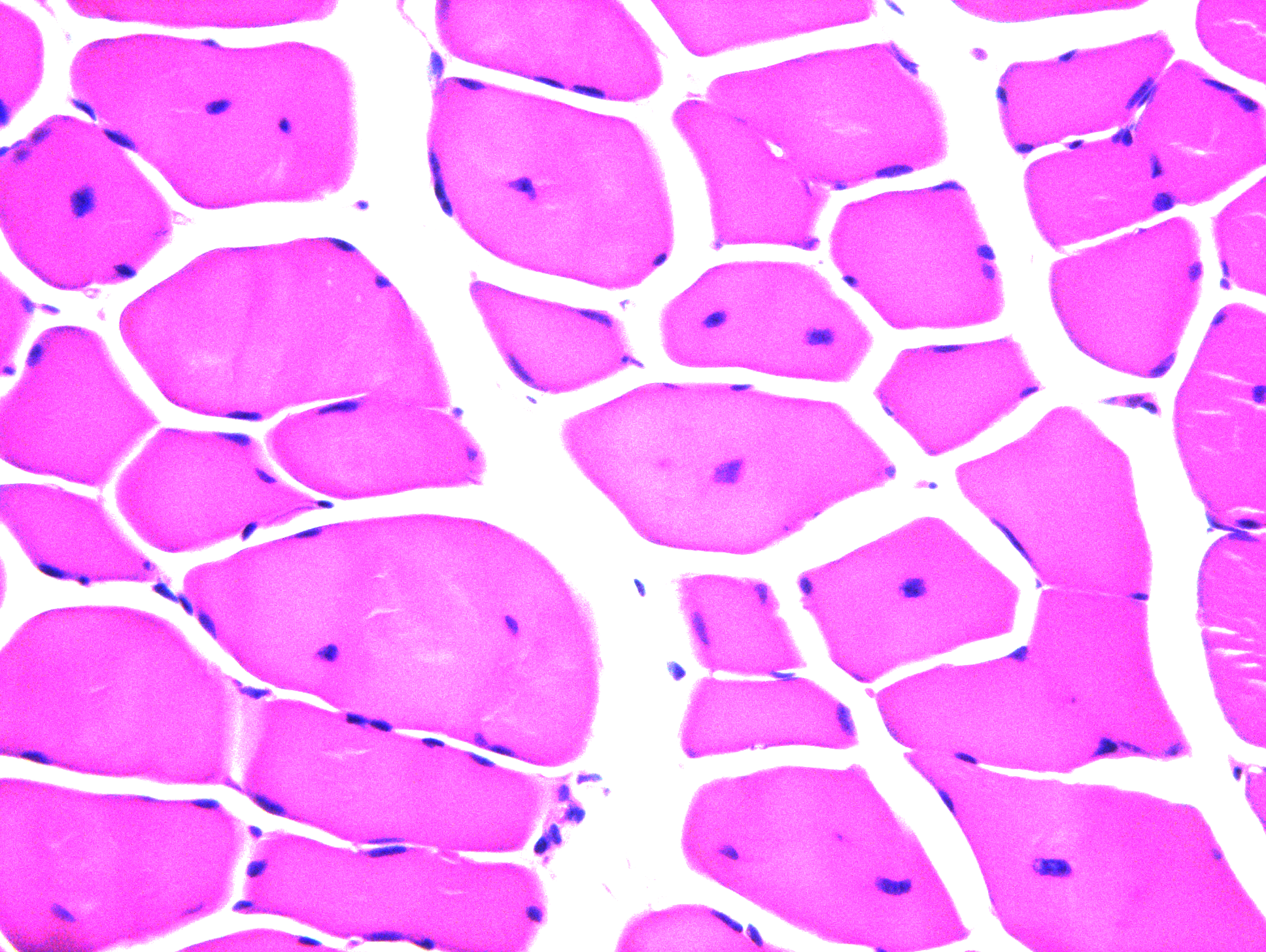

Supplement: Supplementary file 6 — Source Data for Figure 6 [file EMMM-15-e16883-s001.zip › Figure 6/6B/6B.tif]
