# Supplementary material for: Skeletal muscle delimited myopathy and verapamil toxicity in SUR2 mutant mouse models of AIMS
Source: EMBO Mol Med. 2023 May 8;15(6):e16883. doi: 10.15252/emmm.202216883 (PMC10245035; doi:10.15252/emmm.202216883)
Supplement: Supplementary file 7 [file EMMM-15-e16883-s007.pdf]

EMBO Molecular Medicine

Skeletal muscle delimited myopathy and verapamil toxicity in SUR2 mutant mouse models of AIMS

Conor McClenaghan, Maya Mukadam, Jacob Roeglin, Robert Tryon, Manfred Grabner, Anamika Dayal, Gretchen Meyer, and Colin Nichols
DOI: 10.15252/emmm.202216883

Corresponding authors: Conor McClenaghan (conor.mcclenaghan@rutgers.edu) , Colin Nichols (cnichols@wustl.edu)

Review Timeline:

Submission Date:	14th Sep 22
Editorial Decision:	4th Oct 22
Appeal Received:	7th Oct 22
Editorial Decision:	17th Oct 22
Revision Received:	12th Mar 23
Editorial Decision:	6th Apr 23
Revision Received:	10th Apr 23
Accepted:	17th Apr 23

Editor: Lise Roth

Transaction Report:

(Note: With the exception of the correction of typographical or spelling errors that could be a source of ambiguity, letters and reports are not edited. Depending on transfer agreements, referee reports obtained elsewhere may or may not be included in this compilation. Referee reports are anonymous unless the Referee chooses to sign their reports.)

4th Oct 2022

Decision on your manuscript EMM-2022-16883

Dear Dr. McClenaghan,

Thank you for the submission of your manuscript to EMBO Molecular Medicine. We have now received feedback from the three referees who agreed to evaluate your work.

As you will see from the reports below, while referee #3 is overall supportive of the study, referees #1 and #2 are more critical and raise a number of fundamental concerns (including, but not limited to, lack of mechanistic understanding, lack of proper controls, insufficient pharmacological evaluation, ...).

Given the nature of the referees' concerns and the amount of time and work that would be required to address them, and considering that at EMBO Press we encourage one round of revisions only in a reasonable time frame, I am afraid I see little choice but to return the manuscript to you at this point with the decision that we cannot offer to publish it.

I am very sorry to disappoint you in this occasion, and hope that the referees' comments are helpful in your continued work in this area.

Yours sincerely,

Lise Roth

Lise Roth
Senior Editor
EMBO Molecular Medicine

***** Reviewer's comments *****

Referee #1 (Comments on Novelty/Model System for Author):

The technical can be strengthened as per recommendation provided to authors.
The novelty and medical impact are outlined below.
The precision of the gene edited models is underscored.

Referee #1 (Remarks for Author):

Genetically altered mice models were the mainstay of the study employed to gain insight into the myopathic component of the recently reported ABCC-9-related intellectual disability and myopathy syndrome (AIMS). The precision of the gene editing approaches used here, along with the remarkable muscle electrophysiological evaluation, enabled linkage of the SUR2 loss of function (compromising the KATP channel complex) with the skeletal muscle disease phenotype. This finding could guide the further understanding of disease pathobiology. In addition, the authors report lack of therapeutic efficacy and potential aggravated toxicity of the calcium channel blocker, verapamil, in the setting of AIMS, an observation of potential medical significance.

Suggestions:
1. Authors should consider more direct evidence of muscle damage as currently provided muscle histology data seem insufficient to conclude of structural deterioration.
2. The pharmacological evaluation will be further strengthened by a more complete dose-escalation study (to establish the LD50 value). Furthermore, authors may consider prospective electrocardiography acquisition (as terminal bradycardia reported here may not reflect the primary cause of death).
3. A negative cardiovascular hemodynamic effect of verapamil should also be considered.
4. As an alternative aimed to limit the excessive systemic verapamil toxicity, authors may consider the value of local calcium channel blocker administration and/or another calcium channel blocker, such as diltiazem used in skeletal myopathies.

5. For broad readership, authors are encouraged to underscore this work in the context of KATP channelopathies reported across multiple organ systems.

Referee #2 (Comments on Novelty/Model System for Author):

Many of the physiological readouts can be influenced by behavior. Inappropriate controls for CRISPR/Cas9 edited mice. Dubious mathematical formulation. Lack of detailed mechanistic understanding.

Referee #2 (Remarks for Author):

The manuscript attempts to shed light on the specific involvement of skeletal muscle ablation of functioning K-ATPase activity on the pathology of AIMS. The superficial nature of the experiments reported here are largely uninformative and limit my enthusiasm for the work.

General comments:
1) It is difficult to make any interpretations regarding muscle performance in SUR2-STOP mice without CRISPR/Cas9 corrected controls. The possibility and influence of off-target effects by CRISPR/Cas9 gene editing have not been excluded.
2) It is not surprising the Myf5 driven channel ablation showed a similar phenotype given the assays reported here. The hang test is not demanding of the cardiovascular system, and the treadmill test cannot be interpreted as reported (see below). The authors must better design experiments to unequivocally determine the relative contribution of each tissue to the phenotype.
3) Total treadmill distance was recorded but not reported. Rather, the authors use a dubious formula for workload to report running performance. The sum of kinetic and potential energy is total mechanical energy, which is generally conserved. The readout here appears to hinge on differences in the mass of the mouse and belt velocity (of which was under acceleration and is therefore not appropriate). No reports of mouse body weights were given. In any case, no conclusions can be made from this data.
4) The phenotyping methods chosen by the authors may not be sensitive enough to detect differences among genotypes. More objective (and less behavioral) methods are required to make any confident conclusions. The reader is left without any meaningful advances.
5) What is the resting membrane potential of SUR2-STOP myofibers? Without functioning Na,K-ATPase activity the cells are likely to be wildly depolarized. How does this influence the observations made during the patch clamp experiments? Are there any compensatory mechanisms regulating ion flux?
6) The most important finding in the manuscript remains poorly explored. The authors uncover a dramatic effect of Verapamil on mortality in SUR2-STOP mice, which was treated like an afterthought.

Specific comments:
1) Statistical comparisons in the bar graphs and figure legends are not well represented.
2) The authors should clarify the types of induced contractions (e.g., isometric).
3) No genotyping or validation of transgenic mice are given. No evidence of Cre-recombination.
4) Group labeling is confusing. Seeming use of inappropriate controls.
5) It is unclear what the numerical values associated R-R Interval variance represent. ECG tracings do not show a high degree of heart rate variability.
6) No or inappropriate scaling or axis labels in several figures, rendering data uninterpretable.
7) Inconsistent group labeling between manuscript text and figures.
8) Too much use of delta values.

Referee #3 (Comments on Novelty/Model System for Author):

The experiments and model systems were well thought out and the resulting data clarify mechanistic details of AIMS etiology and help guide potential therapeutic approaches for AIMS.

Referee #3 (Remarks for Author):

The authors use both a global and a skeletal muscle-specific mouse model of ABCC9 truncation, which causes loss of function in the SUR2 subunit and therefore loss of function of ATP-sensitive potassium (KATP) channels. Using the mouse models, the authors demonstrate that loss of skeletal muscle KATP channel function underlies the myopathy in AIMS, rather than the cardiac channel loss. The authors also discover that ABCC9 truncation results in abnormal generation of unstimulated forces in skeletal muscle, reflecting aspects of human AIMS. They also find that excessive Ca^{2+} influx through CaV1.1 channels is not responsible for myopathology, and instead discovered that the Ca^{2+} channel blocker verapamil leads to premature death of AIMS mice, likely by bradyarrhythmia/heart block - suggesting against the use of calcium channel blockers to treat the

symptoms of AIMS. The study is well-executed and the manuscript well-written with appropriate and clear figures. I have some minor points that should be addressable by adding clarification in the manuscript:

1) The authors mention they used mice of both sexes for most of the studies, but did not describe if there were sex differences on any of the phenotypes and/or if the sexes were match within groups, and pooled within groups. Please clarify in the manuscript.

2) Please describe any known sex differences in KATP and/or Cav1.1 function in mouse or human cardiac, skeletal muscle or other relevant tissue systems.

3) Are there differences in the role of Cav1.1 in mouse versus human cardiac or skeletal muscle tissue that could underlie potentially different responses to verapamil?

4) How did the authors arrive at the doses of verapamil used in the study? Is it possible that lower doses could be therapeutic without the toxicity? Were the control and STOP475 groups in these experiments matched for sex?

As a service to authors, EMBO provides authors with the possibility to transfer a manuscript that one journal cannot offer to publish to another EMBO publication. The full manuscript and if applicable, reviewers reports are automatically sent to the receiving journal to allow for fast handling and a prompt decision on your manuscript. For more details of this service, and to transfer your manuscript to another EMBO title please click on Link Not Available

Please do not share this URL as it will give anyone who clicks it access to your account.

EMBO Molecular Medicine

***** Reviewer's comments *****

Referee #1 (Comments on Novelty/Model System for Author):
The technical can be strengthened as per recommendation provided to authors.
The novelty and medical impact are outlined below.
The precision of the gene edited models is underscored.

(Remarks for Author):
Genetically altered mice models were the mainstay of the study employed to gain insight into the myopathic component of the recently reported ABCC-9-related intellectual disability and myopathy syndrome (AIMS). The precision of the gene editing approaches used here, along with the remarkable muscle electrophysiological evaluation, enabled linkage of the SUR2 loss of function (compromising the KATP channel complex) with the skeletal muscle disease phenotype. This finding could guide the further understanding of disease pathobiology. In addition, the authors report lack of therapeutic efficacy and potential aggravated toxicity of the calcium channel blocker, verapamil, in the setting of AIMS, an observation of potential medical significance.
We are grateful for the recognition of the novelty and medical impact of out studies and the suggestions for improvements.

Suggestions:
1. Authors should consider more direct evidence of muscle damage as currently provided muscle histology data seem insufficient to conclude of structural deterioration.
We agree that this would be informative. In response, we are performing additional immunohistochemical and gene expression studies to attempt to identify molecular mechanisms of myopathy. The results of these experiments could be included in revision.

2. The pharmacological evaluation will be further strengthened by a more complete dose-escalation study (to establish the LD50 value). Furthermore, authors may consider prospective electrocardiography acquisition (as terminal bradycardia reported here may not reflect the primary cause of death).
Again, we agree that this would be informative, and plan to carry out a more detailed dose-escalation study.

3. A negative cardiovascular hemodynamic effect of verapamil should also be considered.
This is an interesting suggestion. We do not expect there to be any based on published norms, but to be certain, we plan to assess blood pressures in response to acute verapamil treatment in WT and SUR2-STOP mice.

4. As an alternative aimed to limit the excessive systemic verapamil toxicity, authors may consider the value of local calcium channel blocker administration and/or another calcium channel blocker, such as diltiazem used in skeletal myopathies.
This is an astute suggestion, and we agree that further study of other potential calcium channel blockers will be informative. This would be a major undertaking and will constitute follow-up studies.

5. For broad readership, authors are encouraged to underscore this work in the context of KATP channelopathies reported across multiple organ systems.
We will expand the discussion to include consideration of KATP channelopathies.

Referee #2 (Comments on Novelty/Model System for Author):
Many of the physiological readouts can be influenced by behavior. Inappropriate controls for CRISPR/Cas9 edited mice. Dubious mathematical formulation. Lack of detailed mechanistic understanding.
(Remarks for Author):
The manuscript attempts to shed light on the specific involvement of skeletal muscle ablation of functioning K-ATPase activity on the pathology of AIMS. The superficial nature of the experiments reported here are largely uninformative and limit my enthusiasm for the work.
We thank the reviewer for taking the time to evaluate the manuscript. To clarify, however, our studies relate to the ATP-sensitive potassium (KATP) channel, of which SUR2 is a key subunit – not the K-ATPase or Na,K,ATPase pump, as referred to in this review. This is not simply a nomenclature mistake: the reviewer notes - correctly - that "without functioning Na,K-ATPase activity the cells are likely to be wildly depolarized". This represents a fundamental misunderstanding of the manuscript, and is not a result of the way the manuscript is written, as the other referees clearly do understand it.

General comments:
1) It is difficult to make any interpretations regarding muscle performance in SUR2-STOP mice without CRISPR/Cas9 corrected controls. The possibility and influence of off-target effects by CRISPR/Cas9 gene editing have not been excluded.
The suggestion that "CRISPR/Cas9 corrected controls" are needed to make interpretations is an unusual expectation for a genome-edited mouse model. While we appreciate the concern about off-target effects, we have (i) validated the on-target effect of the mutations on KATP channel activity in native cells, and (ii) mitigated the risk of potential off-target effects confounding these studies by extensive outbreeding, and by using littermate wild-type SUR2 control mice in all relevant experiments – the gold standard controls. Therefore, for any off-target mutation to confound the studies, any additional, unknown mutation

would have to be in strong linkage disequilibrium with the intended ABCC9/SUR2 mutation. This is a formal possibility for this and all other studies of genetically modified animal models, but is highly unlikely. Our confidence in our conclusions is bolstered by identifying the key phenotypes (exercise intolerance and verapamil toxicity) in *two distinct* CRISPR mutant SUR2-STOP lines. The likelihood of both being confounded by additional mutations is significantly reduced.

2) It is not surprising the Myf5 driven channel ablation showed a similar phenotype given the assays reported here. The hang test is not demanding of the cardiovascular system, and the treadmill test cannot be interpreted as reported (see below). The authors must better design experiments to unequivocally determine the relative contribution of each tissue to the phenotype.
The possibility of non-skeletal muscle delimited effects of SUR2/KATP loss-of-function on exercise tolerance has been proposed (Stoller et al., 2007). As we discuss, this was a reasonable proposal given the roles of vascular KATP channels in the dynamic perfusion of skeletal muscle. Our studies clearly demonstrate, for the first time, a consequence of KATP channel knockdown in skeletal muscle specifically.

3) Total treadmill distance was recorded but not reported. Rather, the authors use a dubious formula for workload to report running performance. The sum of kinetic and potential energy is total mechanical energy, which is generally conserved. The readout here appears to hinge on differences in the mass of the mouse and belt velocity (of which was under acceleration and is therefore not appropriate). No reports of mouse body weights were given. In any case, no conclusions can be made from this data.
We chose this analysis to maintain consistency with previous analyses from multiple groups (PMID: 12271142, 25648265). However, given the concerns of the referee, we will now additionally report performance in terms of time sustained, and distance travelled, and include measurements of body weight.

4) The phenotyping methods chosen by the authors may not be sensitive enough to detect differences among genotypes. More objective (and less behavioral) methods are required to make any confident conclusions. The reader is left without any meaningful advances.
We are uncertain what more sensitive methods the referee is referring to but would be willing to perform additional phenotyping if justified.

5) What is the resting membrane potential of SUR2-STOP myofibers? Without functioning Na,K-ATPase activity the cells are likely to be wildly depolarized. How does this influence the observations made during the patch clamp experiments? Are there any compensatory mechanisms regulating ion flux?

As noted above, this study is not related to the Na,K-ATPase pump. The referee appears to have misunderstood which protein is in question. The comment regarding the consequence of Na,K-ATPase LoF is valid, but irrelevant. Loss of KATP has previously been shown to be without major effects on membrane potential in resting skeletal muscle, but does cause membrane potential depolarization in fatiguing stimuli, as we discuss.

6) The most important finding in the manuscript remains poorly explored. The authors uncover a dramatic effect of Verapamil on mortality in SUR2-STOP mice, which was treated like an afterthought.
We agree that this is an important finding. It was not an afterthought, but should be considered alongside the preceding data showing that skeletal muscle specifically is a tissue for targeting with therapies.

Specific comments:
1) Statistical comparisons in the bar graphs and figure legends are not well represented.
We would be happy to accommodate any specific suggestions.

2) The authors should clarify the types of induced contractions (e.g., isometric).
We will clarify this in revision.

3) No genotyping or validation of transgenic mice are given. No evidence of Cre-recombination.
Dominant-negative gene expression was clearly demonstrated in patch-clamp studies, in which KATP conductances are abolished in SkM-DN mouse muscle. This is *the* most relevant measure of transgene expression, more important than Cre-recombination, or transcript level, as it demonstrates effects at the protein level. That said, we can incorporate evidence of CRE-recombination in revision.

4) Group labeling is confusing. Seeming use of inappropriate controls.
We would be happy to make appropriate changes in labeling in response to any specific suggestions. The use of inappropriate controls is not clear to us.

5) It is unclear what the numerical values associated R-R Interval variance represent. ECG tracings do not show a high degree of heart rate variability.
We apologize for the absence of units of R-R interval variance, which will be corrected in revision. We disagree that the tracing does not show a variance in R-R interval, there is clearly an increased interval between the 3rd and 4th QRS complex, compared to the preceding intervals.

6) No or inappropriate scaling or axis labels in several figures, rendering data uninterpretable.

We will correct any absent labeling in revision, though are not sure of the specific figures referred to here.

7) Inconsistent group labeling between manuscript text and figures.
We will proof-read for any labeling discrepancies, but again are not sure of specifics from this comment.

8) Too much use of delta values.
Unfortunately, we are not sure what is referred to by "delta values"

Referee #3 (Comments on Novelty/Model System for Author):
The experiments and model systems were well thought out and the resulting data clarify mechanistic details of AIMS etiology and help guide potential therapeutic approaches for AIMS.
(Remarks for Author):
The authors use both a global and a skeletal muscle-specific mouse model of ABCC9 truncation, which causes loss of function in the SUR2 subunit and therefore loss of function of ATP-sensitive potassium (KATP) channels. Using the mouse models, the authors demonstrate that loss of skeletal muscle KATP channel function underlies the myopathy in AIMS, rather than the cardiac channel loss. The authors also discover that ABCC9 truncation results in abnormal generation of unstimulated forces in skeletal muscle, reflecting aspects of human AIMS. They also find that excessive Ca2+ influx through CaV1.1 channels is not responsible for myopathology, and instead discovered that the Ca2+ channel blocker verapamil leads to premature death of AIMS mice, likely by bradyarrhythmia/heart block - suggesting against the use of calcium channel blockers to treat the symptoms of AIMS. The study is well-executed and the manuscript well-written with appropriate and clear figures. I have some minor points that should be addressable by adding clarification in the manuscript:
We are grateful for the positive comments and welcome the suggestions.

1) The authors mention they used mice of both sexes for most of the studies, but did not describe if there were sex differences on any of the phenotypes and/or if the sexes were match within groups, and pooled within groups. Please clarify in the manuscript.
All references to N numbers and sexes will be complete in the revised version. No significant differences were found between male and female mice of the same genotype in any experiment except in treadmill tests of dominant negative mice, where female Myf5-cre+ mice performed worse than males. For this reason, we focused on male mice for this experiment – this will be explained in the revised version.

2) Please describe any known sex differences in KATP and/or Cav1.1 function in mouse or human cardiac, skeletal muscle or other relevant tissue systems.

3) Are there differences in the role of Cav1.1 in mouse versus human cardiac or skeletal muscle tissue that could underlie potentially different responses to verapamil?
We are unaware of any differences but will perform a detailed literature review and include any discussion in revision.

4) How did the authors arrive at the doses of verapamil used in the study? Is it possible that lower doses could be therapeutic without the toxicity? Were the control and STOP475 groups in these experiments matched for sex?
These dosages were based on previous studies (in rats) showing tolerance and blood pressure lowering effects (and therefore in vivo efficacy) of the drug (PMID: 6184556). We show that the dose which causes death in the SUR2-STOP mice is without beneficial effects in the SkM-DN mice, which survive administration, and so do not expect lower doses to be beneficial. This can be tested in the dose escalation study suggested by reviewer 1 however and could be included in revision.

17th Oct 2022

Dear Dr. McClenaghan,

Thank you for your appeal asking us to reconsider our decision on your manuscript.
I have carefully read your letter and have reached out to referee #2 regarding the reference to the wrong channel in his/her report.
Referee #2 replied:
"Indeed, I incorrectly referenced the KATP channel in this particular comment. However, the concern remains: how does knockout of this channel affect resting membrane potential of the isolated myofibers, and are there any compensatory mechanisms. Regardless, the point still should be addressed by the authors. In viewing other comments that raise serious shortcomings of the manuscript, the channel becomes less important per se. For example, the highly behavioral-dependent phenotyping methods used remain a poor choice independent on the specific ion channel studied. Overall, the data presented in the manuscript are not convincing (due to inappropriate controls and questionable reporting methods); and the manuscript suffers from a serious lack of mechanistic understanding."

To reach a balanced decision and as mentioned in a previous correspondence, I have also communicated your provisional point-by-point rebuttal letter to an external advisor. This adviser read your manuscript and the referees' reports, and suggested to allow resubmission of the manuscript after adequate revisions. Therefore, after internal discussion with my colleagues, we decided to reconsider our decision and invite revisions of the manuscript. I will involve an additional referee to evaluate the revised manuscript.

EMBO Molecular Medicine encourages a single round of revision only and therefore, acceptance or rejection of the manuscript will depend on the completeness of your responses included in the next, final version of the manuscript.

When submitting your revised manuscript, please carefully review the instructions that follow below. We perform an initial quality control of all revised manuscripts before re-review; failure to include requested items will delay the evaluation of your revision.

We require:

1) A .docx formatted version of the manuscript text (including legends for main figures, EV figures and tables). Please make sure that the changes are highlighted to be clearly visible.

2) Individual production quality figure files as .eps, .tif, .jpg (one file per figure). For guidance, download the 'Figure Guide PDF' (https://www.embopress.org/page/journal/17574684/authorguide#figureformat).

3) At EMBO Press we ask authors to provide source data for the main and EV figures. Our source data coordinator will contact you to discuss which figure panels we would need source data for and will also provide you with helpful tips on how to upload and organize the files.
Numerical data should be provided as individual .xls or .csv files (including a tab describing the data). For blots or microscopy, uncropped images should be submitted (using a zip archive if multiple images need to be supplied for one panel). Additional information on source data and instruction on how to label the files are available at
.

4) A .docx formatted letter INCLUDING the reviewers' reports and your detailed point-by-point responses to their comments. As part of the EMBO Press transparent editorial process, the point-by-point response is part of the Review Process File (RPF), which will be published alongside your paper.

5) A complete author checklist, which you can download from our author guidelines (https://www.embopress.org/page/journal/17574684/authorguide#submissionofrevisions). Please insert information in the checklist that is also reflected in the manuscript. The completed author checklist will also be part of the RPF.

6) Please note that all corresponding authors are required to supply an ORCID ID for their name upon submission of a revised manuscript.

7) It is mandatory to include a 'Data Availability' section after the Materials and Methods. Before submitting your revision, primary datasets produced in this study need to be deposited in an appropriate public database, and the accession numbers and database listed under 'Data Availability'. Please remember to provide a reviewer password if the datasets are not yet public (see https://www.embopress.org/page/journal/17574684/authorguide#dataavailability).
In case you have no data that requires deposition in a public database, please state so in this section. Note that the Data Availability Section is restricted to new primary data that are part of this study.

8) For data quantification: please specify the name of the statistical test used to generate error bars and P values, the number (n) of independent experiments (specify technical or biological replicates) underlying each data point and the test used to calculate p-values in each figure legend. The figure legends should contain a basic description of n, P and the test applied. Graphs must include a description of the bars and the error bars (s.d., s.e.m.). Please provide exact p values.

9) Our journal encourages inclusion of *data citations in the reference list* to directly cite datasets that were re-used and obtained from public databases. Data citations in the article text are distinct from normal bibliographical citations and should directly link to the database records from which the data can be accessed. In the main text, data citations are formatted as follows: "Data ref: Smith et al, 2001" or "Data ref: NCBI Sequence Read Archive PRJNA342805, 2017". In the Reference list, data citations must be labeled with "[DATASET]". A data reference must provide the database name, accession number/identifiers and a resolvable link to the landing page from which the data can be accessed at the end of the reference. Further instructions are available at .

10) We replaced Supplementary Information with Expanded View (EV) Figures and Tables that are collapsible/expandable online. A maximum of 5 EV Figures can be typeset. EV Figures should be cited as 'Figure EV1, Figure EV2" etc... in the text and their respective legends should be included in the main text after the legends of regular figures.
- For the figures that you do NOT wish to display as Expanded View figures, they should be bundled together with their legends in a single PDF file called *Appendix*, which should start with a short Table of Content. Appendix figures should be referred to in the main text as: "Appendix Figure S1, Appendix Figure S2" etc.
- Additional Tables/Datasets should be labeled and referred to as Table EV1, Dataset EV1, etc. Legends have to be provided in a separate tab in case of .xls files. Alternatively, the legend can be supplied as a separate text file (README) and zipped together with the Table/Dataset file.
See detailed instructions here:

11) The paper explained: EMBO Molecular Medicine articles are accompanied by a summary of the articles to emphasize the major findings in the paper and their medical implications for the non-specialist reader. Please provide a draft summary of your article highlighting
- the medical issue you are addressing,
- the results obtained and
- their clinical impact.
This may be edited to ensure that readers understand the significance and context of the research. Please refer to any of our published articles for an example.

12) For more information: There is space at the end of each article to list relevant web links for further consultation by our readers. Could you identify some relevant ones and provide such information as well? Some examples are patient associations, relevant databases, OMIM/proteins/genes links, author's websites, etc...

13) Author contributions: CRediT has replaced the traditional author contributions section because it offers a systematic machine readable author contributions format that allows for more effective research assessment. Please remove the Authors Contributions from the manuscript and use the free text boxes beneath each contributing author's name in our system to add specific details on the author's contribution. More information is available in our guide to authors.

14) Conflict of interest: We updated our journal's competing interests policy in January 2022 and request authors to consider both actual and perceived competing interests. Please review the policy https://www.embopress.org/competing-interests and update your competing interests if necessary.

15) Every published paper now includes a 'Synopsis' to further enhance discoverability. Synopses are displayed on the journal webpage and are freely accessible to all readers. They include a short stand first (maximum of 300 characters, including space) as well as 2-5 one-sentences bullet points that summarizes the paper. Please write the bullet points to summarize the key NEW findings. They should be designed to be complementary to the abstract - i.e. not repeat the same text. We encourage inclusion of key acronyms and quantitative information (maximum of 30 words / bullet point). Please use the passive voice. Please attach these in a separate file or send them by email, we will incorporate them accordingly.

Please also suggest a striking image or visual abstract to illustrate your article as a PNG file 550 px wide x 300-600 px high.

16) As part of the EMBO Publications transparent editorial process initiative (see our Editorial at http://embomolmed.embopress.org/content/2/9/329), EMBO Molecular Medicine will publish online a Review Process File (RPF) to accompany accepted manuscripts.
In the event of acceptance, this file will be published in conjunction with your paper and will include the anonymous referee reports, your point-by-point response and all pertinent correspondence relating to the manuscript. Let us know whether you agree with the publication of the RPF and as here, if you want to remove or not any figures from it prior to publication.
Please note that the Authors checklist will be published at the end of the RPF.

EMBO Molecular Medicine has a "scooping protection" policy, whereby similar findings that are published by others during review or revision are not a criterion for rejection. Should you decide to submit a revised version, I do ask that you get in touch after three months if you have not completed it, to update us on the status.

I look forward to receiving your revised manuscript.

Yours sincerely,

Lise Roth

Lise Roth, PhD
Senior Editor
EMBO Molecular Medicine

EMBO Molecular Medicine

***** Reviewer's comments *****

Referee #1 (Comments on Novelty/Model System for Author):
The technical can be strengthened as per recommendation provided to authors.
The novelty and medical impact are outlined below.
The precision of the gene edited models is underscored.

(Remarks for Author):
Genetically altered mice models were the mainstay of the study employed to gain insight into the myopathic component of the recently reported ABCC-9-related intellectual disability and myopathy syndrome (AIMS). The precision of the gene editing approaches used here, along with the remarkable muscle electrophysiological evaluation, enabled linkage of the SUR2 loss of function (compromising the KATP channel complex) with the skeletal muscle disease phenotype. This finding could guide the further understanding of disease pathobiology. In addition, the authors report lack of therapeutic efficacy and potential aggravated toxicity of the calcium channel blocker, verapamil, in the setting of AIMS, an observation of potential medical significance.
We are grateful for the recognition of the novelty and medical impact of our studies and the suggestions for improvements.

Suggestions:
1. Authors should consider more direct evidence of muscle damage as currently provided muscle histology data seem insufficient to conclude of structural deterioration.
We thank the reviewer for this suggestion and have added additional histopathological data. These data show that muscle from skeletal muscle dominant-negative (SkM-DN) mice also exhibits necrotic fibers, as observed in HE stained sections, and as further illustrated by macrophage infiltration. Such necrotic fibers were not observed from WT muscle samples. SkM-DN muscle also exhibited increased fibrosis, as determined by picrosirius red staining. Finally, consistent with increased muscle injury response in sedentary mice, we observed increased numbers of Pax7 positive satellite cells in SkM-DN muscle. These new findings have been included in the new Fig 3.

2. The pharmacological evaluation will be further strengthened by a more complete dose-escalation study (to establish the LD50 value). Furthermore, authors may consider prospective electrocardiography acquisition (as terminal bradycardia reported here may not reflect the primary cause of death).
We agree that it would be informative to explore this further. Our data so far at least indicate that the LD50 for verapamil in SUR2-STOP^{475} mice is in between the doses tested

(i.e. between 0.3 g/l and 0.9 g/l in drinking water), which narrows this to a relatively small range. Our findings suggest that detailed further study of the effects of verapamil alongside other calcium channel blockers is warranted and should form the basis of follow up studies (see below). Notably, our ECG analysis did not show any initial obvious bradycardia or arrythmia in the SUR2-STOP mice (Fig. 5D), although the footpad ECG method is probably not sensitive enough to identify subtle abnormalities. Thus, we would like to carry out additional follow up studies (including comparison of verapamil with other calcium channel blockers) using more sophisticated telemetry recording and analysis, although these are not currently available to us.

3. A negative cardiovascular hemodynamic effect of verapamil should also be considered.
This is an interesting suggestion. As we have previously reported (Smeland et al., Nat Comms, 2019), SUR2-STOP mice exhibit higher than normal blood pressures – most likely due to the loss of KATP channel in vascular smooth muscle. Therefore, of course, the naïve prediction would be that verapamil should decrease and potentially normalize blood pressures, via calcium channel blockade in smooth muscle. We cannot rule out unexpected hemodynamic effects, but in a limited experiment (see Fig i below), invasive blood pressure measurement via carotid artery cannulation in isoflurane-anesthetized mice that survived verapamil revealed no drastic hemodynamic effects (SUR2-STOP without verapamil, n = 3; SUR2-STOP with verapamil, n = 2).

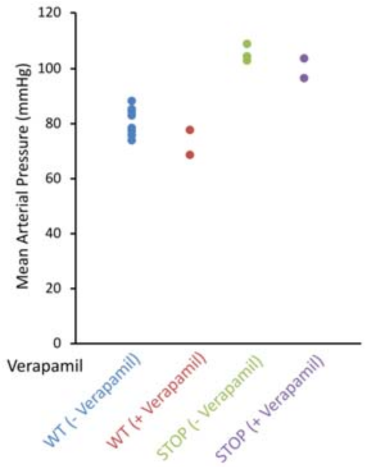

Fig i: Mean arterial blood pressure measured in anesthetized mice via carotid artery catheterization. Verapamil induced a mild decrease in blood pressure in both wild type and SUR2-STOP[475] mice.

4. As an alternative aimed to limit the excessive systemic verapamil toxicity, authors may consider the value of local calcium channel blocker administration and/or another calcium channel blocker, such as diltiazem used in skeletal myopathies.
This is an astute suggestion, and we agree that further study of other potential calcium channel blockers will be informative. This would be a major undertaking and will constitute follow-up studies.

5. For broad readership, authors are encouraged to underscore this work in the context of KATP channelopathies reported across multiple organ systems.
We have expanded the discussion to include consideration of KATP channelopathies, as below:

"Interestingly, exercise intolerance, muscle fatigue, and histopathology are also observed in the rare KATP channelopathy, Cantú Syndrome, which arises from gain-of-function mutations in either ABCC9 (SUR2) or KCNJ8 (Kir6.1)45-47. Whether this is due to intrinsic defects in skeletal muscle physiology or is due to altered cardiovascular function and/or systemic perfusion remains to be established. Individuals with LoF mutations in KCNJ11 (Kir6.2) suffer from congenital hyperinsulinism (CHI). Although muscle fatigue is not typically reported, an extended consanguineous family, exhibiting a syndrome of CHI and rhabdomyolysis has been reported in association with the Kir6.2[R34H] mutation48. In this case, the CHI was severe, and potentially the loss of Kir6.2-dependent KATP was profound: previous analysis of mutation of this arginine residue renders the channels completely insensitive to the essential activator PIP249. Why myopathy is not more widely observed in CHI is not clear but could be explained by incomplete loss-of-function or possibly some underappreciated contribution of Kir6.1 subunits to skeletal muscle channels."

Referee #2 (Comments on Novelty/Model System for Author):
Many of the physiological readouts can be influenced by behavior. Inappropriate controls for CRISPR/Cas9 edited mice. Dubious mathematical formulation. Lack of detailed mechanistic understanding.
(Remarks for Author):
The manuscript attempts to shed light on the specific involvement of skeletal muscle ablation of functioning K-ATPase activity on the pathology of AIMS. The superficial nature of the experiments reported here are largely uninformative and limit my enthusiasm for the work.
We thank the reviewer for taking the time to evaluate the manuscript. To clarify, however, our studies relate to the ATP-sensitive potassium (KATP) channel, of which SUR2 is a key subunit – not the K-ATPase or Na,K,ATPase pump, as referred to in this review. This appears to represent a fundamental misunderstanding of the manuscript, but not one that results from the way the manuscript is written, as the other referees clearly do understand this.

General comments:
1) It is difficult to make any interpretations regarding muscle performance in SUR2-STOP mice without CRISPR/Cas9 corrected controls. The possibility and influence of off-target effects by CRISPR/Cas9 gene editing have not been excluded.

The suggestion that CRISPR/Cas9 corrected controls are needed to make interpretations is an unusual expectation for a genome-edited mouse model. While we appreciate the concern about off-target effects, we have (i) validated the on-target effect of the mutations on KATP channel activity in native cells, and (ii) mitigated the risk of potential off-target effects confounding these studies by extensive outbreeding, and by using littermate wild-type SUR2 control mice in all relevant experiments – the gold standard controls. Therefore, for any off-target mutation to confound the studies, an additional unknown mutation would have to be in strong linkage disequilibrium with the intended ABCC9/SUR2 mutation. This is a formal possibility for this and all other studies of genetically modified animal models, but is highly unlikely. Our confidence in our conclusions is bolstered by identifying the key phenotypes (exercise intolerance and verapamil toxicity) in *two distinct* CRISPR mutant SUR2-STOP lines. The likelihood of both being confounded by additional mutations is significantly reduced.

2) It is not surprising the Myf5 driven channel ablation showed a similar phenotype given the assays reported here. The hang test is not demanding of the cardiovascular system, and the treadmill test cannot be interpreted as reported (see below). The authors must better design experiments to unequivocally determine the relative contribution of each tissue to the phenotype.
The possibility of non-skeletal muscle delimited effects of SUR2/KATP loss-of-function on exercise tolerance has been proposed (Stoller et al., 2007). As we discuss, this was a reasonable proposal given the roles of vascular KATP channels in the dynamic perfusion of skeletal muscle. Our studies clearly demonstrate, for the first time, a consequence of KATP channel knockdown in skeletal muscle specifically.

3) Total treadmill distance was recorded but not reported. Rather, the authors use a dubious formula for workload to report running performance. The sum of kinetic and potential energy is total mechanical energy, which is generally conserved. The readout here appears to hinge on differences in the mass of the mouse and belt velocity (of which was under acceleration and is therefore not appropriate). No reports of mouse body weights were given. In any case, no conclusions can be made from this data.
We chose this analysis to maintain consistency with previous analyses from multiple groups (PMID: 12271142, 25648265). However, given the concerns of the referee, we have substituted figures showing distance travelled on the treadmill in place of the workload figures (new Fig. 1D and Fig, 2D). The treadmill protocol underwent step-wise increases in velocity (increased by 3m/min every 3 minutes). The differences observed definitively did not arise due to differences in the mass of the mice. We have now included data on mouse

weights in the relevant figure legends; there was no significant difference in weight between WT and SUR2-STOP mice, nor for any group used in the dominant-negative studies.

4) The phenotyping methods chosen by the authors may not be sensitive enough to detect differences among genotypes. More objective (and less behavioral) methods are required to make any confident conclusions. The reader is left without any meaningful advances.
We are uncertain what more sensitive methods the referee is referring to as no specific details have been provided.

5) What is the resting membrane potential of SUR2-STOP myofibers? Without functioning Na,K-ATPase activity the cells are likely to be wildly depolarized. How does this influence the observations made during the patch clamp experiments? Are there any compensatory mechanisms regulating ion flux?

As noted above, this study is not related to the Na,K-ATPase pump. The referee appears to have misunderstood which protein is in question, and thus the comment regarding the consequence of Na,K-ATPase LoF is valid, but not relevant. Kir6.2-KO or pharmacological inhibition of KATP has previously been shown to be without major effects on membrane potential in resting skeletal muscle but does cause membrane potential depolarization in fatiguing stimuli (Cifelli et al., 2008, DOI: 10.1113/expphysiol.2008.042572; Zhu et al., 2014, doi: 10.1085/jgp.201311;063). We appreciate the prompt to clarify this and have amendment the text as below:

"KATP channels likely contribute little to determination of the resting membrane potential in resting muscle, but in fatiguing stimuli the activation of functional KATP channels protects isolated myofibers from excessive membrane depolarization, cytosolic Ca2+ overload, and abnormal development of unstimulated tension21-23,31"

6) The most important finding in the manuscript remains poorly explored. The authors uncover a dramatic effect of Verapamil on mortality in SUR2-STOP mice, which was treated like an afterthought.
We agree that this is an important finding. It was not an afterthought, but should be considered alongside the preceding data showing that skeletal muscle specifically is a tissue for targeting with therapies.

Specific comments:
1) Statistical comparisons in the bar graphs and figure legends are not well represented.

We have modified the figures to include p values as per the journal standards. Description of N numbers and p values are now also included in the figure legends, and full description of test statistics have been included with source data files.

2) The authors should clarify the types of induced contractions (e.g., isometric).
Thank you for the suggestion, we have added this.

3) No genotyping or validation of transgenic mice are given. No evidence of Cre-recombination.
Dominant-negative gene expression was clearly demonstrated in patch-clamp studies, which show that KATP conductances are abolished in SkM-DN mouse muscle. This is the most relevant measure of transgene expression, more important than Cre-recombination, or transcript level, as it demonstrates the intended functional effects at the protein level.

4) Group labeling is confusing. Seeming use of inappropriate controls.
We are unsure of the specific issues but have endeavored to label all groups as clearly as possible.

5) It is unclear what the numerical values associated R-R Interval variance represent. ECG tracings do not show a high degree of heart rate variability.
We apologize for the absence of units of R-R interval variance, which has been corrected in revision. We disagree that the tracing does not show a variance in R-R interval, there is clearly an increased interval between the 3rd and 4th QRS complex, compared to the preceding intervals.

6) No or inappropriate scaling or axis labels in several figures, rendering data uninterpretable.
We have added scale bars to histology images.

7) Inconsistent group labeling between manuscript text and figures.
We have additionally proof-read for labeling discrepancies, but again are not sure of specifics from this comment.

8) Too much use of delta values.
Unfortunately, we are not sure what is referred to by “delta values”

Referee #3 (Comments on Novelty/Model System for Author):
The experiments and model systems were well thought out and the resulting data clarify mechanistic details of AIMS etiology and help guide potential therapeutic approaches for AIMS.
(Remarks for Author):

The authors use both a global and a skeletal muscle-specific mouse model of ABCC9 truncation, which causes loss of function in the SUR2 subunit and therefore loss of function of ATP-sensitive potassium (KATP) channels. Using the mouse models, the authors demonstrate that loss of skeletal muscle KATP channel function underlies the myopathy in AIMS, rather than the cardiac channel loss. The authors also discover that ABCC9 truncation results in abnormal generation of unstimulated forces in skeletal muscle, reflecting aspects of human AIMS. They also find that excessive Ca2+ influx through CaV1.1 channels is not responsible for myopathology, and instead discovered that the Ca2+ channel blocker verapamil leads to premature death of AIMS mice, likely by bradyarrhythmia/heart block - suggesting against the use of calcium channel blockers to treat the symptoms of AIMS. The study is well-executed and the manuscript well-written with appropriate and clear figures. I have some minor points that should be addressable by adding clarification in the manuscript:

We are grateful for the positive comments and welcome the suggestions.

1) The authors mention they used mice of both sexes for most of the studies, but did not describe if there were sex differences on any of the phenotypes and/or if the sexes were match within groups, and pooled within groups. Please clarify in the manuscript.
We appreciate the reviewer highlighting this. We did not observe any significant difference between male and female mice of the same genotype in any experiment ($p > 0.05$ according to Mann Whitney U tests comparing male v female of the same genotype), *except* for the treadmill test, where female Myf5+ mice appeared to fatigue more quickly than their male counterparts. Therefore, for the dominant-negative treadmill study we used only male mice for all groups – as now explained more fully in the methods. In all other experiments, where no sex dependent effects were apparent, we combined male and female mice (as now fully described in the figure legends) to maximize statistical power and conserve mice required for studies.

2) Please describe any known sex differences in KATP and/or Cav1.1 function in mouse or human cardiac, skeletal muscle or other relevant tissue systems.

For KATP channels, increased SUR2A transcript levels and Kir6.2 and SUR2A protein levels have been reported in female hearts compared with males; and sex differences in KATP may impact myocardial infarct sizes (Ranki et al. 2001, DOI: 10.1016/s0735-1097(01)01428-0; Johnson et al., 2006, DOI: 10.1152/ajpheart.01291.2005). Importantly, we observed verapamil induced deaths in both male and female mice (7 of 8 male and 4 of 6 female SUR2-STOP^{475} mice died within 28 days of 0.9 g/l verapamil). To our knowledge, sex differences in skeletal muscle KATP channels have not been studied. Subtle sex-dependent differences are possible and warrant further study, but again we saw no significant difference in exercise

tolerance between WT or SUR2-STOP male and female mice (according to Mann Whitney U tests comparing male v female of the same genotype).
For Cav1.1, a recent study showed there was no difference in L-type currents, Ca2+ release, or skeletal muscle EC coupling between male and female mice (Beqollari et al., 2020, DOI: 10.1016/j.bbrc.2019.11.164).

3) Are there differences in the role of Cav1.1 in mouse versus human cardiac or skeletal muscle tissue that could underlie potentially different responses to verapamil?
We are unaware of significant differences between mouse and human $Ca_V1.1$. The IC50 values for inhibition of cardiac Cav channels is in the lower μM range for racemic verapamil in human and rodent model systems and the major amino acids responsible for verapamil binding to L-type Ca2+ channels are highly conserved between mice and humans (Striessnig et al., 1998 DOI: 10.1016/s0165-6147(98)01171-7 ; Fig. 3b).

4) How did the authors arrive at the doses of verapamil used in the study? Is it possible that lower doses could be therapeutic without the toxicity? Were the control and STOP475 groups in these experiments matched for sex?
These doses were based on previous studies (in rats) that showed tolerance and blood pressure lowering effects (and therefore in vivo efficacy) of the drug (PMID: 6184556). We show that the dose which causes death in the SUR2-STOP mice is without beneficial effects in the SkM-DN mice (all ofwhich survive administration), and so we do not expect lower doses to be beneficial. We observed verapamil-induced deaths in both male and female mice (7 of 8 male and 4 of 6 female SUR2-STOP475 mice died within 28 days of 0.9 g/l verapamil).

6th Apr 2023

Dear Dr. McClenaghan,

Thank you for the submission of your revised manuscript to EMBO Molecular Medicine.
This revised version was sent back to referees #1 and #3, and we additionally asked the independent expert who had advised us on your initial submission to review the revised version. We have now received the report from this expert (now referee #4) and referee #3. Referee #1 has not gotten back to us yet, but referee #4 also evaluated your answers to this referee.
As you will see, both referees #3 and #4 are now supportive of publication, and we will therefore be able to accept your manuscript once the following editorial points will be addressed:

1/ Main manuscript text:
- Please address the queries from our data editors in the data edited manuscript file. Please remove the yellow highlights, and only keep in track changes mode any new modification.
- We can accommodate a maximum of 5 keywords, please adjust accordingly.
- Materials and methods:
o Animals: please indicate the housing and husbandry conditions.
o Please provide antibody dilutions.
o Statistics: please include a sentence about randomization, blinding and inclusion/exclusion criteria.
- Data Availability section: this section is present twice in the manuscript, please only keep it after the Material and Methods section ("This study includes no data deposited in external repositories").
- Author contributions: CRediT has replaced the traditional author contributions section because it offers a systematic machine-readable author contributions format that allows for more effective research assessment. Please remove the Authors Contributions from the manuscript and use the free text boxes beneath each contributing author's name in our system to add specific details on the author's contribution. More information is available in our guide to authors.
- Conflict of interest: Please indicate the full section title "Disclosure statement and competing interests".
- Please place the references before the figure legends. References should be listed alphabetically and list 10 authors before "et al.". DOIs should be removed.

2/ Figures: please address the comments from referee #4 on the quality of the H&E images.

3/ Please note that all corresponding authors are required to supply an ORCID ID for their name upon submission of a revised manuscript. An ORCID identifier is currently missing for Colin G Nichols.

4/ Synopsis:
- Thank you for providing a synopsis picture. Please resize it as png/jpeg/tiff file 550px wide x 300-600px high and make sure the text remains legible.
- Please also provide a synopsis text: Synopses are displayed on the journal webpage and are freely accessible to all readers. They include a short stand first (maximum of 300 characters, including space) as well as 2-5 one-sentences bullet points that summarizes the paper. Please write the bullet points to summarize the key NEW findings. They should be designed to be complementary to the abstract - i.e. not repeat the same text. We encourage inclusion of key acronyms and quantitative information (maximum of 30 words / bullet point). Please use the passive voice.

5/ As part of the EMBO Publications transparent editorial process initiative (see our Editorial at http://embomolmed.embopress.org/content/2/9/329), EMBO Molecular Medicine will publish online a Review Process File (RPF) to accompany accepted manuscripts.
This file will be published in conjunction with your paper and will include the anonymous referee reports, your point-by-point response and all pertinent correspondence relating to the manuscript. Let us know whether you agree with the publication of the RPF and as here, if you want to remove or not any figures from it prior to publication.
Please note that the Authors checklist will be published at the end of the RPF.

I look forward to receiving your revised manuscript.

Yours sincerely,

Lise Roth

Lise Roth, PhD

Senior Editor
EMBO Molecular Medicine

***** Reviewer's comments *****

Referee #3 (Remarks for Author):

The authors have satisfactorily addressed my previous concerns.

Referee #4 (Comments on Novelty/Model System for Author):

Please note that I was not the reviewer of the first version, and thus for me this was essentially a novel review. I found the manuscript novel, interesting and well done.

Referee #4 (Remarks for Author):

This is a novel, interesting and well conducted study. The Authors have answered the comments of the Reviewers and I have no further issues.
A minor comment: the quality of the H&E images in Fig. 4A and B is suboptimal, as also the WT sections show abnormal spaces between fibres, usually the result of fixation/processing problems.

Point by point response to referee comments

***** Reviewer's comments *****

Referee #3 (Remarks for Author):

The authors have satisfactorily addressed my previous concerns.

We thank the referee for their input.

Referee #4 (Comments on Novelty/Model System for Author):

Please note that I was not the reviewer of the first version, and thus for me this was essentially a novel review. I found the manuscript novel, interesting and well done.

Referee #4 (Remarks for Author):

This is a novel, interesting and well conducted study. The Authors have answered the comments of the Reviewers and I have no further issues.

A minor comment: the quality of the H&E images in Fig. 4A and B is suboptimal, as also the WT sections show abnormal spaces between fibres, usually the result of fixation/processing problems.

Thank you to the referee for their positive review and constructive comment. We agree that the H&E images show some spacing between myofibers because of the fixation/processing. We also accept that this is suboptimal for presentation, but these fixing effects do not impact the quantification of the centrally nucleated fibers and were present to some extent in all samples subject to this analysis.

17th Apr 2023

Dear Dr. McClenaghan,

Thank you for addressing the queries from our data editors. I am pleased to inform you that your manuscript is accepted for publication and is now being sent to our publisher to be included in the next available issue of EMBO Molecular Medicine!

We would like to remind you that as part of the EMBO Publications transparent editorial process initiative, EMBO Molecular Medicine will publish a Review Process File online to accompany accepted manuscripts. If you do NOT want the file to be published or would like to exclude figures, please immediately inform the editorial office via e-mail.

Please read below for additional IMPORTANT information regarding your article, its publication and the production process.

Congratulations on your interesting work,

With kind regards,

Lise Roth

Lise Roth, Ph.D
Senior Editor
EMBO Molecular Medicine

Follow us on Twitter @EmboMolMed
Sign up for eTOCs at embopress.org/alertsfeeds

*** *** *** IMPORTANT INFORMATION *** *** ***

SPEED OF PUBLICATION
The journal aims for rapid publication of papers, using using the advance online publication "Early View" to expedite the process: A properly copy-edited and formatted version will be published as "Early View" after the proofs have been corrected. Please help the Editors and publisher avoid delays by providing e-mail address(es), telephone and fax numbers at which author(s) can be contacted.

Should you be planning a Press Release on your article, please get in contact with embomolmed@wiley.com as early as possible, in order to coordinate publication and release dates.

LICENSE AND PAYMENT:

All articles published in EMBO Molecular Medicine are fully open access: immediately and freely available to read, download and share.

EMBO Molecular Medicine charges an article processing charge (APC) to cover the publication costs. You, as the corresponding author for this manuscript, should have already received a quote with the article processing fee separately. Please let us know in case this quote has not been received.

Once your article is at Wiley for editorial production you will receive an email from Wiley's Author Services system, which will ask you to log in and will present you with the publication license form for completion. Within the same system the publication fee can be paid by credit card, an invoice, pro forma invoice or purchase order can be requested.

Payment of the publication charge and the signed Open Access Agreement form must be received before the article can be published online.

PROOFS

You will receive the proofs by e-mail approximately 2 weeks after all relevant files have been sent o our Production Office. Please return them within 48 hours and if there should be any problems, please contact the production office at embopressproduction@wiley.com.

Please inform us if there is likely to be any difficulty in reaching you at the above address at that time. Failure to meet our deadlines may result in a delay of publication.

All further communications concerning your paper proofs should quote reference number EMM-2022-16883-V4 and be directed to the production office at embopressproduction@wiley.com.

EMBO Press Author Checklist

McClenaghan
EMBO Molecular Medicine
EMM-2022-16883-V2-Q

USEFUL LINKS FOR COMPLETING THIS FORM
The EMBO Journal - Author Guidelines
EMBO Reports - Author Guidelines
Molecular Systems Biology - Author Guidelines
EMBO Molecular Medicine - Author Guidelines

Reporting Checklist for Life Science Articles (updated January 2022)
This checklist is adapted from Materials Design Analysis Reporting (MDAR) Checklist for Authors. MDAR establishes a minimum set of requirements in transparent reporting in the life sciences (see Statement of Task: 10.31222/osf.io/9sm4x). Please follow the journal's guidelines in preparing your manuscript.
Please note that a copy of this checklist will be published alongside your article.

Abridged guidelines for figures
1. Data
The data shown in figures should satisfy the following conditions:
- the data were obtained and processed according to the field’s best practice and are presented to reflect the results of the experiments in an accurate and unbiased manner.
- ideally, figure panels should include only measurements that are directly comparable to each other and obtained with the same assay.
- plots include clearly labeled error bars for independent experiments and sample sizes. Unless justified, error bars should not be shown for technical replicates.
- if n<5, the individual data points from each experiment should be plotted. Any statistical test employed should be justified.
- Source Data should be included to report the data underlying figures according to the guidelines set out in the authorship guidelines on Data Presentation.

2. Captions
Each figure caption should contain the following information, for each panel where they are relevant:
- a specification of the experimental system investigated (eg cell line, species name).
- the assay(s) and method(s) used to carry out the reported observations and measurements.
- an explicit mention of the biological and chemical entity(ies) that are being measured.
- an explicit mention of the biological and chemical entity(ies) that are altered/varied/perturbed in a controlled manner.
- the exact sample size (n) for each experimental group/condition, given as a number, not a range;
- a description of the sample collection allowing the reader to understand whether the samples represent technical or biological replicates (including how many animals, litters, cultures, etc.).
- a statement of how many times the experiment shown was independently replicated in the laboratory.
- definitions of statistical methods and measures:
  - common tests, such as t-test (please specify whether paired vs. unpaired), simple χ2 tests, Wilcoxon and Mann-Whitney tests, can be unambiguously identified by name only, but more complex techniques should be described in the methods section;
  - are tests one-sided or two-sided?
  - are there adjustments for multiple comparisons?
  - exact statistical test results, e.g., P values = x but not P values < x;
  - definition of ‘center values’ as median or average;
  - definition of error bars as s.d. or s.e.m.

Please complete ALL of the questions below.
Select "Not Applicable" only when the requested information is not relevant for your study.

Materials

Newly Created Materials	Information included in the manuscript?	In which section is the information available? (Reagents and Tools Table, Materials and Methods, Figures, Data Availability Section)
New materials and reagents need to be available; do any restrictions apply?	Yes	Materials and Methods

Antibodies	Information included in the manuscript?	In which section is the information available? (Reagents and Tools Table, Materials and Methods, Figures, Data Availability Section)
For **antibodies** provide the following information:<br>- Commercial antibodies: RRID (if possible) or supplier name, catalogue number and or/clone number<br>- Non-commercial: RRID or citation	Yes	Materials and Methods

DNA and RNA sequences	Information included in the manuscript?	In which section is the information available? (Reagents and Tools Table, Materials and Methods, Figures, Data Availability Section)
**Short novel DNA or RNA including primers, probes**: provide the sequences.	Not Applicable	

Cell materials	Information included in the manuscript?	In which section is the information available? (Reagents and Tools Table, Materials and Methods, Figures, Data Availability Section)
**Cell lines:** Provide species information, strain. Provide accession number in repository **OR** supplier name, catalog number, clone number, and/**OR** RRID.	Not Applicable	
**Primary cultures:** Provide species, strain, sex of origin, genetic modification status.	Not Applicable	
Report if the cell lines were recently **authenticated** (e.g., by STR profiling) and tested for mycoplasma contamination.	Not Applicable	

Experimental animals	Information included in the manuscript?	In which section is the information available? (Reagents and Tools Table, Materials and Methods, Figures, Data Availability Section)
**Laboratory animals or Model organisms:** Provide species, strain, sex, age, genetic modification status. Provide accession number in repository **OR** supplier name, catalog number, clone number, **OR** RRID.	Yes	Materials and Methods
**Animal observed in or captured from the field:** Provide species, sex, and age where possible.	Not Applicable	
Please detail **housing and husbandry conditions**.	Yes	Materials and Methods

Plants and microbes	Information included in the manuscript?	In which section is the information available? (Reagents and Tools Table, Materials and Methods, Figures, Data Availability Section)
**Plants:** provide species and strain, ecotype and cultivar where relevant, unique accession number if available, and source (including location for collected wild specimens).	Not Applicable	
**Microbes:** provide species and strain, unique accession number if available, and source.	Not Applicable	

Human research participants	Information included in the manuscript?	In which section is the information available? (Reagents and Tools Table, Materials and Methods, Figures, Data Availability Section)
If collected and within the bounds of privacy constraints report on age, sex and gender or ethnicity for all study participants.	Not Applicable	

Core facilities	Information included in the manuscript?	In which section is the information available? (Reagents and Tools Table, Materials and Methods, Figures, Data Availability Section)
If your work benefited from core facilities, was their service mentioned in the acknowledgments section?	Yes	Acknowledgements

Design

Study protocol	Information included in the manuscript?	In which section is the information available? (Reagents and Tools Table, Materials and Methods, Figures, Data Availability Section)
If study protocol has been **pre-registered, provide DOI in the manuscript**. For clinical trials, provide the trial registration number **OR** cite DOI.	Not Applicable	
Report the **clinical trial registration number** (at ClinicalTrials.gov or equivalent), where applicable.	Not Applicable	

Laboratory protocol	Information included in the manuscript?	In which section is the information available? (Reagents and Tools Table, Materials and Methods, Figures, Data Availability Section)
Provide DOI OR other citation details if **external detailed step-by-step protocols** are available.	Not Applicable	

Experimental study design and statistics	Information included in the manuscript?	In which section is the information available? (Reagents and Tools Table, Materials and Methods, Figures, Data Availability Section)
Include a statement about **sample size** estimate even if no statistical methods were used.	Not Applicable	
Were any steps taken to minimize the effects of subjective bias when allocating animals/samples to treatment (e.g. **randomization procedure**)? If yes, have they been described?	Yes	Materials and Methods
Include a statement about **blinding** even if no blinding was done.	Yes	Materials and Methods
Describe **inclusion/exclusion criteria** if samples or animals were excluded from the analysis. Were the criteria pre-established?<br><br>If sample or data points were omitted from analysis, report if this was due to attrition or intentional exclusion and provide justification.	Not Applicable	
For every figure, are **statistical tests** justified as appropriate? Do the data meet the assumptions of the tests (e.g., normal distribution)? Describe any methods used to assess it. Is there an estimate of variation within each group of data? Is the variance similar between the groups that are being statistically compared?	Yes	Results, Figures, Figure Legends

Sample definition and in-laboratory replication	Information included in the manuscript?	In which section is the information available? (Reagents and Tools Table, Materials and Methods, Figures, Data Availability Section)
In the figure legends: state number of times the experiment was **replicated** in laboratory.	Yes	Figure Legends
In the figure legends: define whether data describe **technical or biological replicates**.	Yes	Figure Legends

Ethics

Ethics	Information included in the manuscript?	In which section is the information available? (Reagents and Tools Table, Materials and Methods, Figures, Data Availability Section)
Studies involving **human participants**: State details of **authority granting ethics approval** (IRB or equivalent committee(s), provide reference number for approval.	Not Applicable	
Studies involving **human participants**: Include a statement confirming that **informed consent** was obtained from all subjects and that the experiments conformed to the principles set out in the WMA Declaration of Helsinki and the Department of Health and Human Services Belmont Report.	Not Applicable	
Studies involving **human participants:** For publication of **patient photos**, include a statement confirming that consent to publish was obtained.	Not Applicable	
Studies involving experimental **animals**: State details of **authority granting ethics approval** (IRB or equivalent committee(s), provide reference number for approval. Include a statement of compliance with ethical regulations.	Yes	Materials and Methods
Studies involving **specimen and field samples:** State if relevant **permits** obtained, provide details of authority approving study; if none were required, explain why.	Not Applicable	

Dual Use Research of Concern (DURC)	Information included in the manuscript?	In which section is the information available? (Reagents and Tools Table, Materials and Methods, Figures, Data Availability Section)
Could your study fall under dual use research restrictions? Please check biosecurity documents and list of **select agents and toxins** (CDC): https://www.selectagents.gov/sat/list.htm	Not Applicable	
If you used a select agent, is the security level of the lab appropriate and reported in the manuscript?	Not Applicable	
If a study is subject to dual use research of concern regulations, is the name of the **authority granting approval and reference number** for the regulatory approval provided in the manuscript?	Not Applicable	

Reporting

The MDAR framework recommends adoption of discipline-specific guidelines, established and endorsed through community initiatives. Journals have their own policy about requiring specific guidelines and recommendations to complement MDAR.

Adherence to community standards	Information included in the manuscript?	In which section is the information available? (Reagents and Tools Table, Materials and Methods, Figures, Data Availability Section)
State if relevant guidelines or checklists (e.g., **ICMJE, MIBBI, ARRIVE, PRISMA**) have been followed or provided.	Not Applicable	
For **tumor marker prognostic studies,** we recommend that you follow the **REMARK** reporting guidelines (see link list at top right). See author guidelines, under 'Reporting Guidelines'. Please confirm you have followed these guidelines.	Not Applicable	
For **phase II and III randomized controlled trials**, please refer to the **CONSORT** flow diagram (see link list at top right) and submit the CONSORT checklist (see link list at top right) with your submission. See author guidelines, under 'Reporting Guidelines'. Please confirm you have submitted this list.	Not Applicable	

Data Availability

Data availability	Information included in the manuscript?	In which section is the information available? (Reagents and Tools Table, Materials and Methods, Figures, Data Availability Section)
Have **primary datasets** been deposited according to the journal's guidelines (see 'Data Deposition' section) and the respective accession numbers provided in the Data Availability Section?	Not Applicable	
Were **human clinical and genomic datasets** deposited in a public access-controlled repository in accordance to ethical obligations to the patients and to the applicable consent agreement?	Not Applicable	
Are **computational models** that are central and integral to a study available without restrictions in a machine-readable form? Were the relevant accession numbers or links provided?	Not Applicable	
If publicly available data were reused, provide the respective **data citations in the reference list**.	Not Applicable	
